# Factors associated with the occurrence of COVID-19 in the pediatric population in hospital settings: a case-control study

**DOI:** 10.1590/1980-220X-REEUSP-2025-0211en

**Published:** 2025-11-17

**Authors:** Denise Desconsi, Juliane Pagliari Araujo, Marcela Demitto Furtado, Rosângela Aparecida Pimenta, Adriana Valongo Zani

**Affiliations:** 1 Universidade Estadual de Londrina, Londrina, PR, Brazil.; 2 Universidade Estadual de Maringá, Maringá, PR, Brazil.

**Keywords:** Child, Hospitalization, COVID-19, Pandemics, Case-Control Studies

## Abstract

**Objective:**

To analyze factors associated with the occurrence of COVID-19 in the pediatric population in hospital settings.

**Method:**

This was a paired case-control study conducted with medical records of children under 14 years of age. The pediatric population with a positive COVID-19 test was considered a case, and the pediatric population with a negative COVID-19 test was considered a control. For each case, a control was used, totaling 486 medical records. Descriptive analysis, bivariate analysis, and logistic regression were performed.

**Results:**

The variables associated with the occurrence of COVID-19 were brown, black, yellow, and indigenous children, emergency room and Intensive Care Unit admission, use of mask and oxygen catheter, antimicrobials, and corticosteroids. Fever, anorexia, non-eupneic respiratory pattern with saturation between 90% and 95%, cough, runny nose, and comorbidities were associated with the outcome.

**Conclusion:**

Advances by providing information on factors associated with COVID-19 in the hospitalized population under 14 years of age, including place of hospitalization, anorexia, runny nose, comorbidity, and corticosteroid use.

## INTRODUCTION

COVID-19 was discovered in 2019, whose etiological agent is SARS-CoV-2, and presented high transmissibility with spread on a global scale, leading the World Health Organization to declare the COVID-19 pandemic in March 2020^(1)^.

COVID-19 spreads rapidly from person to person, either through direct contact or through exposure to small droplets of infected secretions on surfaces^(2)^. In the pediatric context, children of all ages can contract the disease, as demonstrated in a multicenter cohort that assessed 582 children with COVID-19, in which it was found that the most affected age group is 10 to 18 years old^(3)^.

Clinical and epidemiological characteristics are constantly changing, and the presentation of COVID-19 symptoms in children can be broad and varied, and may be asymptomatic or symptomatic, in addition to presenting severe cases such as severe acute respiratory distress^(4)^.

In a systematic literature review, it was observed that of the 342 children who tested positive for COVID-19, 51 were asymptomatic^(5)^. Data obtained in Wuhan, China, at the beginning of the pandemic, indicate that, of the 171 hospitalized children, with an average age between 6 and 7 years old and who tested positive for COVID-19, 27 were asymptomatic^(6)^. However, it is possible to see that the manifestations of the disease act differently in adults and children, and are often similar to other respiratory viruses, presenting a common clinical presentation, with fever, cough, and headache^(7)^. Furthermore, in a scoping review, the presence of gastrointestinal symptoms presented by the pediatric population with COVID-19 was identified^(8)^.

The scarcity of data on related conditions, symptoms, and COVID-19 in children under 14 years of age indicates gaps that need to be filled^(9)^. This study should help in understanding the variability of the disease and contribute to nursing performance, which plays a prominent role in disease prevention and care.

Therefore, early identification of symptoms in children is necessary, as there are many factors involved that can favor contagion and worsening of the disease, in addition to reducing transmission of the virus^(8)^. Therefore, the question is: what are the factors associated with the occurrence of COVID-19 in the pediatric population in hospital settings?

This study aimed to analyze the factors associated with the occurrence of COVID-19 in the pediatric population in hospital settings.

## METHOD

### Study Design

This is a paired case-control study, described according to the STrengthening the Reporting of OBservational studies in Epidemiology tool.

### Study Site

The study was conducted in the pediatric emergency department, Intensive Care Unit (ICU), and pediatric inpatient unit of a public university hospital (UH) located in the northern region of the state of Paraná, Brazil. The institution is a reference for COVID-19 treatment. This study considered the emergency room, ward, and pediatric ICU as admission locations, given the need to keep children hospitalized in these locations due to demand. COVID-19 was confirmed at the child’s hospitalization location.

### Population

The study population included (n = 486) medical records of pediatric patients, under 14 years of age, treated at the UH and who were admitted to the hospital between June 2020 and December 2022. This period was understood as the peak of the COVID-19 pandemic in Brazil, and for pediatric hospitalizations, the study hospital considers the neonatal population from 0 to 28 days, and the child population, from 29 days to 14 years.

### Selection Criteria and Sample Definition

The pediatric population with positive COVID-19 was considered a case. The control group consisted of the pediatric population with symptoms but who tested negative for COVID-19. A control group was performed for each case^(10)^, resulting in 243 cases and 243 controls, totaling 486 medical records/patients. Therefore, all cases of pediatric patients positive for COVID-19 during the period under investigation were used, eliminating the need for sample size calculation. To pair the samples between cases and controls, data affinity between individuals was used, taking into account characteristics but not those under investigation. Thus, to define the control group in reference to the case group, sex and age variables were observed, seeking similarity between the populations.

**Figure 1:**
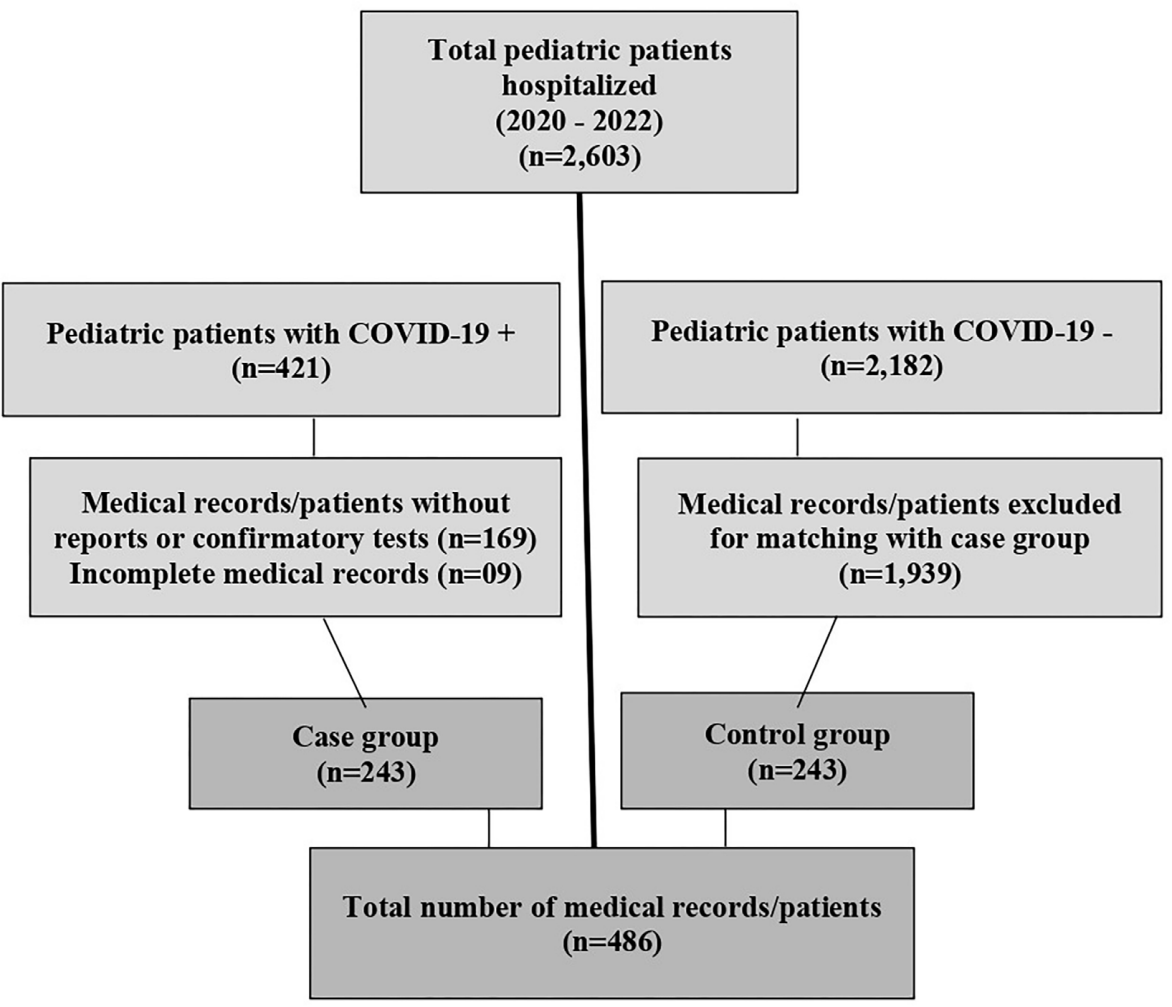
Selection of medical records/patients for the study. Londrina, PR, Brazil, 2023.

Children who did not have confirmatory laboratory tests for COVID-19 were excluded. To identify the sample, two spreadsheets provided by the hospital’s health surveillance unit were used: the first is a spreadsheet of patients with a positive COVID-19 test; and the second is a spreadsheet of patients with a negative COVID-19 test. Figure 1 presents the flowchart for selecting medical records/patients and presents case and control groups, considering inclusion and exclusion criteria.

### Study Variables

The outcome variable was COVID-19. Positivity was confirmed by reverse transcription-PCR, followed by polymerase chain reaction, antigen, serology, and rapid immunoglobulin G and M serology tests, which were recorded in patients’ medical records and on the COVID-19 spreadsheet.

Exposure variables were sex, age, race/ethnicity, city of origin, hospital admission, place of admission, length of stay, ventilatory support, antimicrobial use, and corticosteroid use. Variables related to signs and symptoms were fever, anorexia, reflux, nausea, vomiting, diarrhea, intestinal bleeding, abdominal pain, respiratory pattern, oxygen saturation, cough, runny nose, and comorbidities. The variables analyzed in this study were supported by previous studies^(5,11,12)^.

### Data Collection

Demographic, clinical, and symptom-related information was extracted from patients’ medical records and entered into two separate electronic spreadsheets. Data collection took place between April 10, 2023, and May 24, 2023, using Medview^®^, the hospital’s electronic medical record system.

### Data Analysis

Data were analyzed using the Statistical Package for the Social Sciences version 26.0. Descriptive analysis included a study sample description, with an emphasis on demographic and clinical characteristics. The absolute and relative frequencies of the variables of interest were measured. Bivariate and multivariate analyses were used to assess the association between the outcome and exposure variables.

In bivariate analysis, Pearson’s chi-square test was applied, and variables with alpha < 0.20, the recommended cut-off point for exploratory analyses of associated factors, were selected for the next stage. Unadjusted Odds Ratios (ORs) and their 95% Confidence Intervals (95%CIs) were calculated.

Multiple models were obtained using binary logistic regression using the backward method, i.e., the order in which exposure variables were removed from the model was determined by the highest significance value. Variables with alpha < 0.05 remained in the model. Adjusted ORs and their 95%CIs were calculated. Goodness-of-fit was measured using the Hosmer-Lesmeshow test, in which a higher p-value indicates a better fit. An alpha < 0.05 was considered statistically significant.

### Ethical Aspects

The study was approved by the Research Ethics Committee of a public educational institution, under Certificate of Presentation for Ethical Consideration 31528920.9.0000.5231 and Opinion 4.415.191. There was no direct contact with patients; therefore, the use of an Informed Consent Form was waived.

## RESULTS

The sample consisted of 486 medical records/patients, of which 243 were confirmed COVID-19 cases and 243 were controls. It was found that 61.3% of children, regardless of the group, were male, under 10 years of age (74.5% case and 77% control), and white (60.9% case and 70.9% control), and were referred from other hospital services (85.6% case and 86.4% control). Regarding the place of hospitalization, children with COVID-19 were hospitalized mainly in the emergency room (47.7%), while those in the control group were mostly in the pediatric ward (66.3%). The median length of hospital stay was three days for cases and one day for controls.

Concerning ventilatory support, 74.5% of children with COVID-19 did not require any device, while in the control group, this percentage was 53.9%. Regarding the use of antimicrobials and corticosteroids, both were more frequent in the control group, administered to 70.4% and 82.7% of children, respectively. Among COVID-19 cases, the use of these medications was lower, with 49.8% of children receiving antimicrobials and 48.1% receiving corticosteroids.

The most prevalent symptoms were fever (60.1% case and 65.8% control), cough (73.3% case and 55.1% control), and runny nose (56.4% case and 74.5% control). Anorexia was more frequent in the case group (56.4%) than in the control group (36.6%). Chronic lung diseases and prematurity were the most common comorbidities (Table 1).

**Table 1:** Demographic and clinical characterization of the hospitalized pediatric population (n = 486) – Londrina, PR, Brazil, 2023.

Variables	Case n (%)	Control n (%)
**Sex**		
Female	94 (38.7)	94 (38.7)
Male	149 (61.3)	149 (61.3)
**Age**		
< 10 years	181 (74.5)	187 (77.0)
≥ 10 years	62 (25.5)	56 (23.0)
Median (IQR)	3.00 (9.00)	4.00 (8.00)
Minimum-Maximum	0–14	0–14
**Race/color**		
White	148 (60.9)	172 (70.9)
Brown, black, yellow, indigenous	95 (39.1)	71 (29.1)
**Hospital admission**		
Referenced	208 (85.6)	210 (86.4)
Spontaneous search	35 (14.4)	33 (13.6)
**Place of hospitalization**		
Emergency room	116 (47.7)	46 (18.9)
Intensive Care Unit	21 (8.7)	36 (18.8)
Pediatric inpatient care	106 (43.6)	161 (66.3)
**Length of hospital stay (days)**		
≥ 7 days	29 (11.9)	30 (12.3)
< 7 days	214 (88.1)	213 (87.7)
Median (IQR)	3.00 (3.00)	1.00 (3.00)
Minimum-Maximum	1–35	1–30
**Ventilatory support**		
Mask	8 (3.3)	25 (10.3)
Catheter	35 (14.4)	65 (26.7)
Orotracheal intubation	19 (7.8)	22 (9.1)
None	181 (74.5)	131 (53.9)
**Antimicrobial use**		
Yes	121 (49.8)	171 (70.4)
No	122 (50.2)	72 (29.6)
**Use of corticosteroids**		
Yes	117 (48.1)	201 (82.7)
No	126 (51.9)	42 (17.3)
**Presence of fever**		
Yes	146 (60.1)	160 (65.8)
No	97 (39.9)	83 (34.2)
**Anorexia**		
Yes	137 (56.4)	89 (36.6)
No	106 (43.6)	154 (63.4)
**Gastric reflux**		
Yes	4 (1.6)	6 (2.5)
No	239 (98.4)	237 (97.5)
**Nausea**		
Yes	25 (10.3)	21 (8.6)
No	218 (89.7)	222 (91.4)
**Vomiting**		
Yes	58 (23.9)	46 (18.9)
No	185 (76.1)	197 (81.1)
**Diarrhea**		
Yes	35 (14.4)	38 (15.6)
No	208 (85.6)	205 (84.4)
**Intestinal bleeding**		
Yes	8 (3.3)	8 (3.3)
No	235 (96.7)	235 (96.7)
**Abdominal pain**		
Yes	44 (18.1)	28 (11.5)
No	199 (81.9)	215 (85.5)
**Breathing pattern**		
Dyspneic	110 (45.3)	67 (27.6)
Tachypneic	9 (3.7)	17 (7.0)
Eupneic	124 (51.0)	159 (65.4)
**Oxygen saturation**		
< 90%	20 (8.2)	22 (9.1)
90 to 95%	97 (39.9)	59 (24.3)
> 95%	126 (51.9)	162 (66.6)
**Cough**		
Yes	178 (73.3)	134 (55.1)
No	65 (26.7)	109 (44.9)
**Runny nose**		
Yes	137 (56.4)	181 (74.5)
No	106 (43.6)	62 (25.5)
**Comorbidities**		
Prematurity	14 (5.8)	20 (8.2)
Chronic lung disease	35 (14.4)	14 (5.8)
Hypoxic-ischemic encephalopathy	10 (4.1)	2 (0.8)
Endocrine diseases	13 (5.3)	2 (0.8)
Obesity	2 (0.8)	3 (1.2)
No comorbidities	169 (69.6)	202 (83.2)

Legend: IQR – interquartile range.

In bivariate analysis, some variables showed a statistically significant association with the occurrence of COVID-19. Children classified as brown, black, yellow, or indigenous were 1.55 times more likely to be diagnosed with COVID-19 than white children (OR = 1.55; 95%CI: 1.1–2.27; p = 0.022). Being admitted to the emergency room almost fourfold increased the chance of COVID-19, compared to pediatric hospitalization (OR = 3.83; 95%CI: 2.52–5.83; p < 0.001). The use of an oxygen mask (OR = 0.23), catheter (OR = 0.38), antimicrobials (OR = 0.42), and corticosteroids (OR = 0.19) was associated with a lower chance of being diagnosed with COVID-19, all with p < 0.001 (Table 2).

**Table 2:** Associations between demographic and clinical factors and the occurrence of COVID-19 in the hospitalized pediatric population (n = 486) – Londrina, PR, Brazil, 2023.

Variables	COVID-19		p-value*	Unadjusted OR (95%CI)
	Case n (%)	Control n (%)		
**Age**				
< 10 years	181 (74.5)	187 (77.0)	0.526	0.87 (0.57–1.32)
≥ 10 years	62 (25.5)	56 (23.0)		
Median (IQR)	3 (8.00)			
Minimum-Maximum	0–14			
**Sex**				
Female	94 (38.7)	94 (38.7)	1.00	1.00 (0.69–1.44)
Male	149 (61.3)	149 (61.3)		
**Race/color**				
White	148 (60.9)	172 (70.8)		
Brown, black, yellow, indigenous	95 (39.1)	71 (29.2)	0.022	1.55 (1.1–2.27)
**Hospital admission**				
Referenced	209 (86.0)	209 (86.0)	1.00	1.00 (0.77–1.29)
Spontaneous search	34 (14.0)	34 (14.0)		
**Place of hospitalization**				
Emergency room	116 (47.7)	46 (18.9)	<0.001	3.83 (2.52–5.83)
Intensive Care Unit	21 (8.6)	36 (14.8)	0.688	0.89 (0.49–1.60)
Pediatric inpatient care	106 (43.7)	161 (66.3)		
**Length of hospital stay (days)**				
≥ 7 days	29 (11.9)	30 (12.3)	0.890	1.04 (0.60–1.79)
< 7 days	214 (88.1)	213 (87.7)		
Median (IQR)	2 (3.00)			
Minimum-Maximum	1–35			
**Ventilatory support**				
Mask	8 (3.3)	25 (10.3)	<0.001	0.23 (0.10–0.53)
Catheter	35 (14.4)	65 (26.7)	<0.001	0.38 (0.24–0.62)
Tube	19 (7.8)	22 (9.1)	0.078	0.62 (0.32–1.20)
Ambient air	181 (74.5)	131 (53.9)		
**Antimicrobial use**				
Yes	121 (49.8)	171 (70.4)	<0.001	0.42 (0.28–0.61)
No	122 (50.2)	72 (29.6)		
**Corticosteroid use**				
Yes	117 (48.1)	201 (82.7)	<0.001	0.19 (0.13–0.24)
No	126 (51.9)	42 (17.3)		

Legend: *Pearson’s chi-square test; OR - Odds Ratio; 95%CI - 95% Confidence Interval; IQR - interquartile range.

Regarding signs and symptoms, children with COVID-19 were more likely to have fever (OR = 1.90; 95%CI: 1.31–2.76; p = 0.001) and anorexia (OR = 2.24; 95%CI: 1.55–3.22; p < 0.001) than the control group. On the other hand, symptoms such as runny nose (OR = 0.44), cough (OR = 0.52), and non-eupneic respiratory pattern (OR = 0.45) were more associated with the control group. The presence of comorbidities was also more common among the control group (32.9% case and 14.4% control; OR = 0.34; p < 0.001), suggesting a lower association with COVID-19 (Table 3).

**Table 3:** Associations between signs and symptoms associated with the occurrence of COVID-19 in the hospitalized pediatric population (n = 486) – Londrina, PR, Brazil, 2023.

Variables	COVID-19		p-value*	Unadjusted OR (95%CI)
	Case n (%)	Control n (%)		
**Presence of fever**				
Yes	171 (70.4)	135 (55.6)	0.001	1.90 (1.31–2.76)
No	72 (29.6)	108 (44.4)		
**Anorexia**				
Yes	137 (56.4)	89 (36.6)	<0.001	2.24 (1.55–3.22)
No	106 (43.6)	154 (63.4)		
**Gastric reflux**				
Yes	5 (2.1)	5 (2.1)	1.00	1.00 (0.29–3.50)
No	238 (97.9)	238 (97.9)		
**Nausea**				
Yes	25 (10.3)	21 (8.6)	0.535	1.21 (0.66–2.23)
No	218 (89.7)	222 (91.4)		
**Vomiting**				
Yes	48 (19.8)	56 (23.0)	0.376	0.82 (0.53–1.27)
No	195 (80.2)	187 (77.0)		
**Diarrhea**				
Yes	41 (16.9)	32 (13.2)	0.253	1.34 (0.81–2.21)
No	202 (83.1)	211 (86.8)		
**Intestinal bleeding**				
Yes	7 (2.9)	9 (3.7)	0.611	0.77 (0.28–2.10)
No	236 (97.1)	234 (96.3)		
**Abdominal pain**				
Yes	42 (17.3)	30 (12.3)	0.125	1.48 (0.89–2.46)
No	201 (82.7)	213 (87.7)		
**Breathing pattern**				
Non-eupneic	78 (32.1)	125 (51.4)	<0.001	0.45 (0.31–0.64)
Eupneic	165 (67.9)	118 (48.6)		
**Oxygen saturation**				
< 90%	21 (8.6)	21 (8.6)	0.309	0.71 (0.37–1.37)
90 to 95%	54 (22.2)	102 (42.0)	<0.001	0.38 (0.25–0.57)
> 95%	168 (69.1)	120 (49.4)		
**Cough**				
Yes	138 (56.8)	174 (71.6)	0.001	0.52 (0.36–0.76)
No	105 (43.2)	69 (28.4)		
**Runny nose**				
Yes	137 (56.4)	181 (74.5)	<0.001	0.44 (0.30–0.65)
No	106 (43.6)	62 (25.5)		
**Comorbidities**				
Yes	35 (14.4)	80 (32.9)	<0.001	0.34 (0.22–0.52)
No	208 (85.6)	163 (67.1)		

Legend: *Pearson’s chi-square test; OR - Odds Ratio; 95%CI - 95% Confidence Interval; IQR - interquartile range.

In multivariate analysis, some variables remained associated with COVID-19 diagnosis in the pediatric population. Children hospitalized in the emergency room were 2.78 times more likely to have COVID-19 (95%CI: 1.33–5.84; p = 0.007), and those in the ICU were 2.52 times more likely (95%CI: 1.54–4.16; p < 0.001), compared to pediatric hospitalization.

The use of corticosteroids increased the chance of COVID-19 by 3.37 times (95%CI: 2.05–5.53; p < 0.001), and the presence of runny nose, by 2.25 times (95%CI: 1.43–3.54; p < 0.001). Having comorbidities was also associated with the outcome (OR = 1.86; 95%CI: 1.12–3.10; p = 0.015). Anorexia, although significant in bivariate analysis, showed an inverse association in the regression, reducing the chance of COVID-19 (OR = 0.32; 95%CI: 0.21–0.49; p < 0.001) (Table 4).

**Table 4:** Logistic regression of demographic, clinical, and sign and symptom variables associated with the occurrence of COVID-19 in the hospitalized pediatric population (n = 486) – Londrina, PR, Brazil, 2023.

Variables	Adjusted OR	95%CI	p-value^‡^
**Place of hospitalization**			
Emergency room	2.78	1.33–5.84	0.007
Intensive Care Unit	2.52	1.54–4.16	0.000
Pediatric inpatient care	1.00		
**Use of corticosteroids**			
Yes	3.37	2.05–5.53	<0.001
No	1.00		
**Anorexia**			
Yes	0.32	0.21–0.49	<0.001
No			
**Runny nose**			
Yes	2.25	1.43–3.54	<0.001
No			
**Comorbidities**			
Yes	1.86	1.12–3.10	0.015
No			

Legend: OR – Odds Ratio; 95%CI – 95% Confidence Interval

^‡^Pearson’s chi-square test.

## DISCUSSION

This study provided evidence on predisposing factors and associations with the occurrence of COVID-19 in the hospitalized pediatric population. Among the indicators used in multivariate analysis, location of hospitalization (emergency room or ICU), corticosteroid use, runny nose, and comorbidities were the most strongly associated with the chance of children contracting COVID-19.

Analysis of results identified that, regardless of the group, there was a higher incidence of COVID-19 in male children. Early research in Wuhan, China, at the beginning of the pandemic, validated this finding, and evidence suggests that male patients tend to have a higher incidence of the disease and, when compared to female patients, are more likely to die or develop severe forms of the disease^(6,13)^.

Among children diagnosed with COVID-19, 74.5% were under 10 years of age. This data supports a study that presents a retrospective analysis of SARS-CoV-2 infections involving 2,135 patients, with a median age of 7 years, making this population susceptible to infection by the virus^(14)^.

The location of hospitalization stood out as a relevant factor, as children admitted to the emergency room were 2.78 times more likely to be diagnosed with COVID-19, while those admitted to the ICU were 2.52 times more likely, compared to those admitted to a pediatric ward. These findings may be related to the initial clinical profile of patients, the higher turnover and overcrowding of beds in these units, and the greater exposure to symptomatic patients, especially at the beginning of the pandemic, when personal protective equipment was scarce and protocols were still being developed^(15)^.

The median length of hospital stay was two days, a result close to that found in the literature^(16)^. The longer the length of hospital stay, the greater the risks for patients due to exposure to infectious agents during hospitalization. Since the pediatric population has the right to companions, their families are also exposed to contracting diseases and, likewise, spreading them^(17)^. The pediatric patient profile is diverse and inherent to the stages of development, and there are some common characteristics regarding hospital care demands to be assigned. This data can be of great relevance in assisting with hospital routines and reorganization of pediatric services, when necessary, along with information on patients’ clinical aspects^(16)^.

It is important to note that 64.2% of hospitalized children did not use ventilatory support. Studies emphasize the need for discussion about the use of ventilatory support to prevent future harm and increased hospitalizations^(18)^. It is known that children are susceptible to developing respiratory tract infections due to their anatomical, physiological and immunological qualities, and when the use of ventilatory support is necessary, the ventilatory weaning process comprises 40% of length of hospitalization^(19)^.

The use of antimicrobials and corticosteroids was widely used in the children studied. In this sample, the analysis indicated that children who used antimicrobials were 58% less likely to develop COVID-19. This use may be justified by the attempt to prevent respiratory tract infections, since, at the beginning of the pandemic, knowledge about the virus was limited, and in the case of respiratory infections, there is the possibility of mixed infection or secondary bacterial infection^(20)^. Another explanation is the empirical coverage for possible superinfection in the respiratory tract that is acquired in a hospital^(21)^.

Corticosteroid use was also associated with a higher risk of COVID-19, with a 3.37-fold increase in the chance of diagnosis compared to controls. However, corticosteroid use is often used in respiratory tract infections due to its anti-inflammatory effect, reduction of viral replication, and regulation of angiotensin-converting enzyme 2 gene expression^(22)^.

In this regard, assessing the description of the signs and symptoms of the pediatric population observed in the study made it possible to verify that the presence of fever was associated with a 90% increase in the chance of a COVID-19 diagnosis. Fever was present in most children in the case group, being one of the most common complaints in children’s clinics and hospitals^(23)^, in addition to being an isolated sign reported in approximately 20 to 30% of pediatric consultations. Furthermore, cough was observed in both groups, which is in line with the study that also highlighted cough as the main symptom identified in 83% of children^(24)^.

Symptoms of cough, runny nose, non-eupneic breathing pattern, and oxygen saturation of 90 to 95% were inversely associated with a diagnosis of COVID-19, being associated with a lower chance of this outcome occurring in the study population. Overall, in most cases of flu-like illness, cough and runny nose were identified as the initial symptoms, considered classic symptoms of airborne infections, and may be followed by other symptoms. Therefore, medications are widely used due to the discomfort they cause and the short-term impact on quality of life^(25)^.

Another important factor identified was the presence of comorbidities, which increased the chance of COVID-19 by 1.86 times. Children with preexisting medical conditions, such as chronic lung disease or a history of prematurity, may be more susceptible to infection and have a less favorable clinical outcome. According to The International Study of Asthma and Allergies in Childhood, a validated and standardized international protocol that supports studies on asthma and allergic diseases, Brazil has a high prevalence of lung diseases, especially asthma and allergic rhinitis^(26)^.

Prematurity still impacts newborn morbidity and mortality and has permanent consequences for child development. In Brazil, from 2011 to 2021, approximately 31,625,722 live births were reported; of these, 3,503,085 were premature, accounting for a prevalence of 11%^(27)^. These consequences affect children’s future, representing a risk factor for those exposed to respiratory viral pathogens. However, our findings reveal that 76.3% of children had no other related illnesses. However, the presence of comorbidities was associated with an 86% increased risk of COVID-19. A possible explanation is that having comorbidities increases the chance of developing an unfavorable prognosis^(15)^.

Among children diagnosed with COVID-19, there was a higher frequency of individuals who self-identified as brown, black, yellow, or indigenous compared to white children. The analysis revealed that these children were 55% more likely to be included in the case group, indicating a statistically significant association between race/color and the occurrence of COVID-19 in the study population. A case-control study and a systematic review with meta-analysis report that racial and ethnic groups (Black, Latino, and Hispanic) are disproportionately affected, possibly due to economic factors, inequalities, and similar health conditions^(14)^.

The use of an oxygen mask and nasal cannula was more frequently associated with the control group. These findings suggest that the use of these ventilatory supports may be related to respiratory conditions of other etiologies. Nasal cannula is one of the most effective non-invasive therapy methods due to its ability to prevent aerosolization and cross-contamination, in addition to being comfortable^(28)^. When ventilatory support is provided, there is a reduction in the body’s effort, aiding in patients’ improvement and restoring normal oxygen levels necessary for health^(29)^.

The results showed that the location of admission (emergency room or ICU), the presence of comorbidities, corticosteroid use, and a runny nose remained significantly associated with a COVID-19 diagnosis. Children admitted to the emergency room were 2.78 times more likely to be diagnosed, those admitted to the ICU were 2.52 times more likely, and those who used corticosteroids were 3.37 times more likely. Furthermore, the presence of a runny nose was associated with a 2.25-fold increase in the chance of being included in the case group. These findings highlight the complexity of healthcare for children affected by COVID-19 and reinforce the need to expand prevention strategies, early diagnosis, and specific therapeutic care for this population group.

Limitations of this study include its retrospective nature, which precluded direct observation of children, and the fact that it was conducted at a single teaching hospital, which may limit the generalizability of results. Another limitation is the lack of blinding of the control group patients by the principal investigator and incomplete information in the database, leading to inconsistency and, consequently, data exclusion.

## CONCLUSION

Among the factors associated with the occurrence of COVID-19 in hospitalized children under 14, the location of admission (emergency room or ICU), corticosteroid use, having a runny nose, and comorbidities were the most strongly associated with disease diagnosis. The results of this study advance knowledge by providing important information on the signs and symptoms presented by children during hospitalization for COVID-19, as well as the factors associated with these occurrences.

## DATA AVAILABILITY

The complete dataset supporting the findings of this study is available within the article itself.

## References

[1] World Health Organization. (2020). Coronavirus disease (COVID-19) pandemic [Internet]..

[2] Götzinger F, Santiago-García B, Noguera-Julián A, Lanaspa M, Lancella L, Calò Carducci FI (2020). COVID-19 in children and adolescents in Europe: a multinational,
multicentre cohort study.. Lancet Child Adolesc Health..

[3] Deville JG, Song E, Ouellette CP (2021). COVID-19: clinical manifestations and diagnosis in
children.. UpToDate [Internet]..

[4] Nehab MF, Menezes LA, Portela MC, Reis LGC, Lima SML (2022). Covid-19: challenges for the organization and repercussions on health
systems and services..

[5] Patel NA (2020). Pediatric COVID-19: systematic review of the
literature.. Am J Otolaryngol..

[6] Lu X, Zhang L, Du H, Zhang J, Li YL, Qu J (2020). Chinese Pediatric Novel Coronavirus Study Team. SARS-CoV-2
infection in children.. N Engl J Med..

[7] Stokes JR, Bacharier LB (2020). Prevention and treatment of recurrent viral-induced wheezing in
the preschool child.. Ann Allergy Asthma Immunol..

[8] Desconsi D, Araujo PJ, Furtado MD, Pimenta AR, Zani VA (2024). Relationship between gastrointestinal symptoms and COVID-19
infection in the pediatric population: a scoping review.. Rev Esc Enferm USP..

[9] Bellino S, Punzo O, Rota CM, Del Manso M, Urdiales MA, Andrianou X (2020). COVID-19 disease severity risk factors for pediatric patients in
Italy.. Pediatrics..

[10] Gordis L (2017). Epidemiologia [Internet]..

[11] Prata-Barbosa A, Lima-Setta F, Santos RG, Lanziotti SV, Castro VER, Souza DC (2020). Pediatric patients with COVID-19 admitted to intensive care units
in Brazil: a prospective multicenter study.. J Pediatr (Rio J)..

[12] Maciel NLE, Gomes CC, Almada LG, Medeiros FN, Cardoso AO, Jabor MP (2021). COVID-19 in children, adolescents and young people: survey in
Espírito Santo, Brazil, 2020.. Epidemiol Serv Saude..

[13] Silva PR, Morais AC, Miranda JOF, Andrade KVF, Santos DV, Martins AL (2024). Prevalence of severe covid-19 cases and associated factors in a
pediatric hospital.. Rev. Baiana Enferm..

[14] Bernardino FB, Alencastro LC, Silva RA, Ribeiro AD, Castilho GR, Gaíva MA (2021). Epidemiological profile of children and adolescents with
COVID-19: a scoping review.. Rev Bras Enferm..

[15] Schmidt CJ, Moraes MA, Goulart SC, Lapa J, Krum BN, Becker RG (2023). Acompanhamento de um ano de crianças hospitalizadas com COVID-19:
estudo prospectivo de coorte.. J Bras Pneumol..

[16] Grunewald STF, Aroeira I, Paiva L, Rossi M (2019). Clinical and demographic profile of the pediatric ward in a
University Hospital.. Resid. Pediatr..

[17] Correia A, Graça D, Caldeira E, Guerreiro G (2021). COVID-19: the resolution of the IPO Lisbon Pediatric
Unit.. Onco..

[18] Patel M, Chowdhury J, Mills N, Marron R, Gangemi A, Dorey-Stein Z (2021). Utility of the ROX index in predicting intubation for patients
with COVID-19: related hypoxemic respiratory failure receiving high-flow
nasal therapy: retrospective cohort study.. JMIRx Med..

[19] Castro Ribeiro A, Estevam Artagoitia R (2021). Ventilatory support in pediatric UTI: observational
study.. Braz J Global Health..

[20] Miqueletto JA, Santos A, Castellano GC, Marcondes L, Lenhani BE, Batista J (2022). Bacterial profile, antimicrobial resistance and secondary
infections in patients with covid-19: an integrative review.. Arch Health Sci..

[21] Spernovasilis NA, Kofteridis DP (2021). COVID-19 and antimicrobial stewardship: what is the
interplay?. Infect Control Hosp Epidemiol..

[22] Kounis NG, Kouni SN, Mplani V, Koniari I (2022). Corticosteroids for mild COVID-19 treatment: opening the
floodgates of therapeutic benefits.. QJM..

[23] Pitoli PJ, Duarte BK, Fragoso AA, Damaceno DG, Sanches Marin MJ (2021). Fever in children: parents’ search for urgent and emergency
services.. Cien Saude Colet..

[24] Mohammad S, Korn K, Schellhaas B, Neurath MF, Goertz RS (2019). Clinical characteristics of influenza in season 2017/2018 in a
German emergency department: a retrospective analysis.. Microbiol Insights..

[25] Gu X, Cao B (2021). In-hospital complications associated with
COVID-19.. Lancet..

[26] Silva MSE, Traebert J, Silva Fo DJ, Traebert E (2023). Prevalence of allergic rhinitis symptoms and associated factors
in six-year-old children in a municipality in southern
Brazil.. Rev Bras Epidemiol..

[27] Alberton M, Rosa VM, Iser BPM (2023). Prevalence and temporal trend of prematurity in Brazil before and
during the COVID-19 pandemic: a historical time series analysis,
2011-2021.. Epidemiol Serv Saude..

[28] Koga Y, Kaneda K, Mizuguchi I, Nakahara T, Miyauchi T, Fujita M (2016). Extent of pleural effusion on chest radiograph is associated with
failure of high-flow nasal cannula oxygen therapy.. J Crit Care..

[29] Marini JJ, Gattinoni L (2020). Management of COVID-19 respiratory distress.. JAMA..

