# Supplementary material for: Factors associated with the occurrence of COVID-19 in the pediatric population in hospital settings: a case-control study
Source: Rev Esc Enferm USP. 2025 Nov 17;59:e20250211. doi: 10.1590/1980-220X-REEUSP-2025-0211en (PMC12662595; doi:10.1590/1980-220X-REEUSP-2025-0211en)
Supplement: Translation

# Translation: Fatores associados à ocorrência da COVID-19 na população pediátrica no contexto hospitalar: estudo caso-controle

**DOI:** 10.1590/1980-220X-REEUSP-2025-0211pt

**Authors:** Denise Desconsi, Juliane Pagliari Araujo, Marcela Demitto Furtado, Rosângela Aparecida Pimenta, Adriana Valongo Zani

**Affiliations:** 1 Universidade Estadual de Londrina, Londrina, PR, Brasil.; 2 Universidade Estadual de Maringá, Maringá, PR, Brasil.

**Keywords:** Criança, Hospitalização, COVID-19, Pandemias, Estudos de Casos e Controles

## Abstract

**Objetivo:**

Analisar os fatores associados à ocorrência da COVID-19 na população pediátrica no contexto hospitalar.

**Método:**

Trata-se de estudo caso-controle pareado, realizado com prontuários de crianças menores de 14 anos. Considerou-se caso a população pediátrica com teste positivo para COVID-19, e controle, a população pediátrica negativa para COVID-19. Para cada caso, utilizou-se um controle, totalizando 486 prontuários. Realizaram-se análise descritiva, análise bivariada e regressão logística.

**Resultados:**

As variáveis associadas à ocorrência da COVID-19 foram crianças pardas, pretas, amarelas e indígenas, internamento em pronto-socorro e Unidade de Terapia Intensiva, uso de máscara e cateter de oxigênio, antimicrobiano, e corticoide. Febre, anorexia, padrão respiratório não eupneico com saturação entre 90% e 95%, presença de tosse, coriza, e ter comorbidade tiveram associação com o desfecho.

**Conclusão:**

Avança ao fornecer informações sobre os fatores associados à COVID-19 na população menor de 14 anos hospitalizada, sendo esses o local de internação, anorexia, coriza, comorbidade e uso de corticoide.

## INTRODUÇÃO

A COVID-19 foi descoberta no ano de 2019, cujo agente etiológico é o SARS-CoV-2, e apresentou alta transmissibilidade com disseminação em escala global, levando a Organização Mundial da Saúde a declarar pandemia de COVID-19 em março de 2020^(1)^.

A transmissão da COVID-19 ocorre de forma rápida de pessoa para pessoa, seja por contato direto ou pela exposição a pequenas gotículas de secreções infectadas em superfície^(2)^. No contexto pediátrico, crianças de todas as idades podem contrair a doença, conforme demonstrado em coorte multicêntrica que avaliou 582 crianças com COVID-19, na qual verificou-se que a faixa etária mais afetada é a de 10 a 18 anos^(3)^.

As características clínicas e epidemiológicas estão em constante mudança, e a apresentação da sintomatologia da COVID-19 nas crianças pode ser ampla e variada, podendo ser assintomática ou sintomática, além de apresentar casos graves como desconforto respiratório agudo grave^(4)^.

Observa-se, em revisão sistemática da literatura, que das 342 crianças positivas para COVID-19, 51 eram assintomáticas^(5)^. Dados obtidos em Wuhan, China, no início da pandemia, apontam que, das 171 crianças internadas, com média de idade entre 6 e 7 anos e com teste positivo para COVID-19, 27 eram assintomáticas^(6)^. Contudo, é possível ver que as manifestações da doença agem diferentemente em adultos e crianças, e muitas vezes é semelhante a outros vírus respiratórios, apresentando uma clínica comum, com febre, tosse e cefaleia^(7)^. Ainda, em revisão de escopo, foi identificado a presença de sintomas gastrointestinais apresentados pela população pediátrica com COVID-19^(8)^.

A escassez de dados sobre as condições relacionadas, sintomas apresentados e a COVID-19 nas crianças menores de 14 anos indica lacunas a serem preenchidas^(9)^. Este estudo deve auxiliar no entendimento da variabilidade da doença e contribuir com o desempenho da enfermagem, a qual atua com destaque na prevenção e cuidado na doença.

Assim, a identificação precoce dos sintomas em crianças é necessária, pois há muitos fatores implicados que podem favorecer o contágio e o agravo da doença, além de diminuir a transmissão do vírus^(8)^. Para tanto, questiona-se: quais os fatores associados à ocorrência da COVID-19 na população pediátrica no contexto hospitalar?

O objetivo deste estudo é analisar os fatores associados à ocorrência da COVID-19 na população pediátrica no contexto hospitalar.

## MÉTODO

### Tipo de Estudo

Trata-se de estudo caso-controle pareado, descrito de acordo com a ferramenta *STrengthening the Reporting of OBservational studies in Epidemiology*.

### Local de Estudo

O estudo foi realizado nos setores de pronto atendimento pediátrico, Unidade de Terapia Intensiva (UTI) e internação pediátrica de hospital universitário (HU) público localizado na região norte do estado do Paraná, Brasil. A instituição é referência para o tratamento da COVID-19. Este estudo considerou como locais de internação o pronto-socorro, enfermaria e UTI pediátrica, visto a necessidade de manter as crianças hospitalizadas nesses locais devido à demanda. A confirmação da COVID-19 deu-se no local de internação da criança.

### População

A população do estudo incluiu (n=486) prontuários de pacientes pediátricos, menores de 14 anos, atendidos no HU e que deram entrada no hospital no período de junho de 2020 a dezembro de 2022. Tal período foi compreendido como pico pandêmico da COVID-19 no Brasil, e para as internações pediátricas, o hospital de estudo considera a população neonatal de 0 a 28 dias, e a infantil, de 29 dias a 14 anos.

### Critérios de Seleção e Definição da Amostra

Foi considerado caso a população pediátrica com COVID-19 positivo. Para o grupo controle, foi considerada a população pediátrica, com sintomas, mas que testou negativo para COVID-19, sendo realizado, para cada caso, um controle^(10)^, obtendo, assim, 243 casos e 243 controles, totalizando 486 prontuários/pacientes. Portanto, usaram-se todos os casos de pacientes pediátricos positivos para COVID-19 no período investigado, não necessitando, assim, de cálculo amostral. Para realizar o pareamento das amostras entre caso e controle, optou-se pela afinidade de dados entre indivíduos, levando características em consideração, porém não aquelas que estavam em investigação. Assim, para realizar a definição do grupo controle em referência ao grupo caso, observaram-se as variáveis sexo e idade, buscando similaridade entre as populações.

Foram excluídas as crianças que não possuíam exames laboratoriais confirmatórios para COVID-19. Para identificar a amostra, foram utilizadas duas planilhas disponibilizadas pelo núcleo de vigilância em saúde da instituição hospitalar: a primeira é uma planilha de pacientes com teste de COVID-19 positivo; e a segunda é uma planilha paciente com teste de COVID-19 negativo. A Figura 1 apresenta o fluxograma de seleção dos prontuários/pacientes, e apresenta os grupos caso e controle, considerando os critérios de inclusão e exclusão.

**Figura 1:** Seleção dos prontuários/pacientes para o estudo. Londrina, PR, Brasil, 2023.

### Variáveis do Estudo

A variável desfecho foi a COVID-19. A positividade foi confirmada por meio de exames de transcrição reversa, seguidos de reação em cadeia da polimerase, antígeno, sorologia, rápido sorológico de imunoglobulina G e M registrados no prontuário dos pacientes e na planilha COVID-19.

As variáveis de exposição foram sexo, idade, raça/cor/etnia, cidade de origem, entrada hospitalar, local de internação, tempo de internação, suporte ventilatório, uso de antimicrobiano e uso corticoides. As variáveis relacionadas aos sinais e sintomas foram presença de febre, anorexia, refluxo, náusea, vômito, diarreia, sangramento intestinal, dor abdominal, padrão respiratório, saturação de oxigênio, tosse, coriza e comorbidades. As variáveis analisadas neste estudo foram apoiadas em estudos realizados previamente^(5,11,12)^.

### Coleta de Dados

As informações demográficas, clínicas e relacionadas aos sinais e sintomas dos pacientes foram extraídas dos prontuários e digitadas em duas planilhas eletrônicas separadamente. A coleta de dados ocorreu entre 10 de abril de 2023 e 24 de maio de 2023, por meio do *Medview* ^®^ *,* sistema informatizado de prontuário eletrônico do hospital.

### Análise de Dados

Os dados foram analisados no pacote estatístico *Statistical Package for the Social Sciences*, versão 26.0. A análise descritiva incluiu a descrição da amostra estudada com ênfase nas características demográficas e clínicas. Foram medidas as frequências absoluta e relativa das variáveis de interesse. Para medir a associação entre o desfecho e as variáveis de exposição, foram utilizadas a análise bivariada e multivariada.

Na análise bivariada, foi aplicado o teste qui-quadrado de Pearson, sendo selecionadas para a próxima etapa as variáveis que apresentaram alfa <0,20, ponto de corte recomendado para análises exploratórias de fatores associados. Foram calculados as Razões de Chance (ou *Odds Ratio* (OR)) brutas e seus Intervalos de Confiança de 95% (IC95%).

Os modelos múltiplos foram obtidos por meio da Regressão Logística Binária, pelo método de *backward*, ou seja, a ordem de retirada das variáveis de exposição no modelo é determinada pelo maior valor de significância. Continuavam no modelo as variáveis com alfa <0,05. Foram calculados os OR ajustados e seus IC95%. A bondade da qualidade do ajuste foi mensurada mediante o teste de Hosmer-Lesmeshow, em que, quanto maior o valor de *p*, melhor é o ajuste. Considerou-se alfa <0,05 como estatisticamente significante.

### Aspectos Éticos

O estudo foi aprovado pelo Comitê de Ética em Pesquisa Envolvendo Seres Humanos de uma instituição pública de ensino, sob Certificado de Apresentação para Apreciação Ética n^o^ 31528920.9.0000.5231 e Parecer n^o^ 4.415.191. Não houve contato direto com os pacientes; portanto, houve a dispensa do uso do Termo de Consentimento Livre e Esclarecido.

## RESULTADOS

A amostra foi constituída por 486 prontuários/pacientes, sendo 243 casos confirmados para COVID-19 e 243 controles. Verificou-se que 61,3% das crianças independentemente do grupo eram do sexo masculino, com idade inferior a 10 anos (74,5% caso e 77% controle) e brancas (60,9% caso e 70,9% controle), e foram referenciadas de outros serviços hospitalares (85,6% caso e 86,4% controle). Quanto ao local de internação, as crianças com COVID-19 foram hospitalizadas principalmente no pronto-socorro (47,7%), enquanto as do grupo controle estavam majoritariamente em enfermaria pediátrica (66,3%). A mediana do tempo de internação foi de três dias, para os casos, e um dia, para os controles.

Em relação ao suporte ventilatório, observou-se que 74,5% das crianças com COVID-19 não necessitaram de nenhum dispositivo, enquanto que, no grupo controle, esse percentual foi de 53,9%. Quanto ao uso de antimicrobianos e corticoides, ambos foram mais frequentes no grupo controle, sendo administrados em 70,4% e 82,7% das crianças, respectivamente. Entre os casos de COVID-19, o uso desses medicamentos foi menor, sendo que 49,8% das crianças receberam antimicrobianos, e 48,1%, corticoides.

Os sintomas mais prevalentes foram febre (60,1% caso e 65,8% controle), tosse (73,3% caso e 55,1% controle) e coriza (56,4% caso e 74,5% controle). A anorexia foi mais frequente no grupo caso (56,4%) do que no controle (36,6%). Doenças pulmonares crônicas e prematuridade foram as comorbidades mais comuns (Tabela 1).

**Tabela 1:** Caracterização demográfica e clínica da população pediátrica hospitalizada (n = 486) – Londrina, PR, Brasil, 2023.

Variáveis	Caso n (%)	Controle n (%)
**Sexo**		
Feminino	94 (38,7)	94 (38,7)
Masculino	149 (61,3)	149 (61,3)
**Idade**		
< 10 anos	181 (74,5)	187 (77,0)
≥ 10 anos	62 (25,5)	56 (23,0)
Mediana (IIQ)	3,00 (9,00)	4,00 (8,00)
Mínimo-Máximo	0–14	0–14
**Raça/cor**		
Branca	148 (60,9)	172 (70,9)
Parda, preta, amarela, indígena	95 (39,1)	71 (29,1)
**Entrada hospitalar**		
Referenciado	208 (85,6)	210 (86,4)
Procura espontânea	35 (14,4)	33 (13,6)
**Local de internação**		
Pronto-socorro	116 (47,7)	46 (18,9)
Unidade de Terapia Intensiva	21 (8,7)	36 (18,8)
Internação pediátrica	106 (43,6)	161 (66,3)
**Tempo de internação (dias)**		
≥ 7 dias	29 (11,9)	30 (12,3)
< 7 dias	214 (88,1)	213 (87,7)
Mediana (IIQ)	3,00 (3,00)	1,00 (3,00)
Mínimo-Máximo	1–35	1–30
**Suporte ventilatório**		
Máscara	8 (3,3)	25 (10,3)
Cateter	35 (14,4)	65 (26,7)
Intubação orotraqueal	19 (7,8)	22 (9,1)
Nenhum	181 (74,5)	131 (53,9)
**Uso de antimicrobiano**		
Sim	121 (49,8)	171 (70,4)
Não	122 (50,2)	72 (29,6)
**Uso de corticoide**		
Sim	117 (48,1)	201 (82,7)
Não	126 (51,9)	42 (17,3)
**Presença de febre**		
Sim	146 (60,1)	160 (65,8)
Não	97 (39,9)	83 (34,2)
**Anorexia**		
Sim	137 (56,4)	89 (36,6)
Não	106 (43,6)	154 (63,4)
**Refluxo gástrico**		
Sim	4 (1,6)	6 (2,5)
Não	239 (98,4)	237 (97,5)
**Náusea**		
Sim	25 (10,3)	21 (8,6)
Não	218 (89,7)	222 (91,4)
**Vômito**		
Sim	58 (23,9)	46 (18,9)
Não	185 (76,1)	197 (81,1)
**Diarreia**		
Sim	35 (14,4)	38 (15,6)
Não	208 (85,6)	205 (84,4)
**Sangramento intestinal**		
Sim	8 (3,3)	8 (3,3)
Não	235 (96,7)	235 (96,7)
**Dor abdominal**		
Sim	44 (18,1)	28 (11,5)
Não	199 (81,9)	215 (85,5)
**Padrão respiratório**		
Dispneico	110 (45,3)	67 (27,6)
Taquipneico	9 (3,7)	17 (7,0)
Eupneico	124 (51,0)	159 (65,4)
**Saturação de oxigênio**		
< 90%	20 (8,2)	22 (9,1)
90 a 95%	97 (39,9)	59 (24,3)
> 95%	126 (51,9)	162 (66,6)
**Tosse**		
Sim	178 (73,3)	134 (55,1)
Não	65 (26,7)	109 (44,9)
**Coriza**		
Sim	137 (56,4)	181 (74,5)
Não	106 (43,6)	62 (25,5)
**Comorbidades**		
Prematuridade	14 (5,8)	20 (8,2)
Doença pulmonares crônicas	35 (14,4)	14 (5,8)
Encefalopatia hipóxico-isquêmica	10 (4,1)	2 (0,8)
Doenças endócrinas	13 (5,3)	2 (0,8)
Obesidade	2 (0,8)	3 (1,2)
Sem comorbidades	169 (69,6)	202 (83,2)

Legenda: IIQ – intervalo interquartílico.

Na análise bivariada, algumas variáveis apresentaram associação estatisticamente significativa com a ocorrência da COVID-19. Crianças classificadas como parda, preta, amarela ou indígena apresentaram 1,55 vezes mais chance de diagnóstico de COVID-19 do que crianças brancas (OR = 1,55; IC95%: 1,1–2,27; p = 0,022). Estar internado no pronto-socorro aumentou em quase quatro vezes a chance de COVID-19, em comparação à internação pediátrica (OR = 3,83; IC95%: 2,52–5,83; p < 0,001). O uso de máscara de oxigênio (OR = 0,23), cateter (OR = 0,38), antimicrobianos (OR = 0,42) e corticoides (OR = 0,19) esteve associado à menor chance de diagnóstico de COVID-19, todos com p < 0,001 (Tabela 2).

**Tabela 2:** Associações entre os fatores demográficos e clínicos e a ocorrência da COVID-19 na população pediátrica hospitalizada (n = 486) – Londrina, PR, Brasil, 2023.

Variáveis	COVID-19		Valor de p*	OR bruto (IC95%)
	Caso n (%)	Controle n (%)		
**Idade**				
< 10 anos	181 (74,5)	187 (77,0)	0,526	0,87 (0,57–1,32)
≥ 10 anos	62 (25,5)	56 (23,0)		
Mediana (IIQ)	3 (8,00)			
Mínimo-Máximo	0–14			
**Sexo**				
Feminino	94 (38,7)	94 (38,7)	1,00	1,00 (0,69–1,44)
Masculino	149 (61,3)	149 (61,3)		
**Raça/cor**				
Branca	148 (60,9)	172 (70,8)		
Parda, preta, amarela, indígena	95 (39,1)	71 (29,2)	0,022	1,55 (1,1–2,27)
**Entrada hospitalar**				
Referenciado	209 (86,0)	209 (86,0)	1,00	1,00 (0,77–1,29)
Procura espontânea	34 (14,0)	34 (14,0)		
**Local de internação**				
Pronto-socorro	116 (47,7)	46 (18,9)	<0,001	3,83 (2,52–5,83)
Unidade de Terapia Intensiva	21 (8,6)	36 (14,8)	0,688	0,89 (0,49–1,60)
Internação pediátrica	106 (43,7)	161 (66,3)		
**Tempo de internação (dias)**				
≥ 7 dias	29 (11,9)	30 (12,3)	0,890	1,04 (0,60–1,79)
< 7 dias	214 (88,1)	213 (87,7)		
Mediana (IIQ)	2 (3,00)			
Mínimo-Máximo	1–35			
**Suporte ventilatório**				
Máscara	8 (3,3)	25 (10,3)	<0,001	0,23 (0,10–0,53)
Cateter	35 (14,4)	65 (26,7)	<0,001	0,38 (0,24–0,62)
Tubo	19 (7,8)	22 (9,1)	0,078	0,62 (0,32–1,20)
Ar ambiente	181 (74,5)	131 (53,9)		
**Uso de antimicrobiano**				
Sim	121 (49,8)	171 (70,4)	<0,001	0,42 (0,28–0,61)
Não	122 (50,2)	72 (29,6)		
**Uso corticoide**				
Sim	117 (48,1)	201 (82,7)	<0,001	0,19 (0,13–0,24)
Não	126 (51,9)	42 (17,3)		

Legenda: *teste qui-quadrado de Pearson; OR - *Odds Ratio*; IC95% - Intervalo de Confiança de 95%; IIQ - intervalo interquartílico.

Em relação aos sinais e sintomas, crianças com COVID-19 apresentaram maior chance de ter febre (OR = 1,90; IC95%: 1,31–2,76; p = 0,001) e anorexia (OR = 2,24; IC95%: 1,55–3,22; p < 0,001) do que o grupo controle. Por outro lado, sintomas como coriza (OR = 0,44), tosse (OR = 0,52) e padrão respiratório não eupneico (OR = 0,45) estavam mais associados ao grupo controle. A presença de comorbidades também foi mais comum entre o grupo controle (32,9% caso e 14,4% controle; OR = 0,34; p < 0,001), sugerindo menor associação com COVID-19 (Tabela 3).

**Tabela 3:** Associações entre os sinais e sintomas associados à ocorrência da COVID-19 na população pediátrica hospitalizada (n = 486) – Londrina, PR, Brasil, 2023.

Variáveis	COVID-19		Valor de p*	OR bruto (IC95%)
	Caso n (%)	Controle n (%)		
**Presença de febre**				
Sim	171 (70,4)	135 (55,6)	0,001	1,90 (1,31–2,76)
Não	72 (29,6)	108 (44,4)		
**Anorexia**				
Sim	137 (56,4)	89 (36,6)	<0,001	2,24 (1,55–3,22)
Não	106 (43,6)	154 (63,4)		
**Refluxo gástrico**				
Sim	5 (2,1)	5 (2,1)	1,00	1,00 (0,29–3,50)
Não	238 (97,9)	238 (97,9)		
**Náusea**				
Sim	25 (10,3)	21 (8,6)	0,535	1,21 (0,66–2,23)
Não	218 (89,7)	222 (91,4)		
**Vômito**				
Sim	48 (19,8)	56 (23,0)	0,376	0,82 (0,53–1,27)
Não	195 (80,2)	187 (77,0)		
**Diarreia**				
Sim	41 (16,9)	32 (13,2)	0,253	1,34 (0,81–2,21)
Não	202 (83,1)	211 (86,8)		
**Sangramento intestinal**				
Sim	7 (2,9)	9 (3,7)	0,611	0,77 (0,28–2,10)
Não	236 (97,1)	234 (96,3)		
**Dor abdominal**				
Sim	42 (17,3)	30 (12,3)	0,125	1,48 (0,89 – 2,46)
Não	201 (82,7)	213 (87,7)		
**Padrão respiratório**				
Não eupneico	78 (32,1)	125 (51,4)	<0,001	0,45 (0,31–0,64)
Eupneico	165 (67,9)	118 (48,6)		
**Saturação de oxigênio**				
< 90%	21 (8,6)	21 (8,6)	0,309	0,71 (0,37–1,37)
90 a 95%	54 (22,2)	102 (42,0)	<0,001	0,38 (0,25–0,57)
> 95%	168 (69,1)	120 (49,4)		
**Tosse**				
Sim	138 (56,8)	174 (71,6)	0,001	0,52 (0,36–0,76)
Não	105 (43,2)	69 (28,4)		
**Coriza**				
Sim	137 (56,4)	181 (74,5)	<0,001	0,44 (0,30–0,65)
Não	106 (43,6)	62 (25,5)		
**Comorbidades**				
Sim	35 (14,4)	80 (32,9)	<0,001	0,34 (0,22–0,52)
Não	208 (85,6)	163 (67,1)		

Legenda: *teste qui-quadrado de Pearson; OR – *Odds Ratio*; IC95% – Intervalo de Confiança de 95%; IIQ – intervalo interquartílico.

Na análise multivariada, algumas variáveis permaneceram associadas ao diagnóstico de COVID-19 na população pediátrica. Crianças hospitalizadas no pronto-socorro apresentaram 2,78 vezes mais chance de terem COVID-19 (IC95%: 1,33–5,84; p = 0,007), e aquelas na UTI, 2,52 vezes mais chance (IC95%: 1,54–4,16; p < 0,001), em comparação à internação pediátrica.

O uso de corticoides aumentou a chance de COVID-19 em 3,37 vezes (IC95%: 2,05–5,53; p < 0,001), e a presença de coriza, em 2,25 vezes (IC95%: 1,43–3,54; p < 0,001). Ter comorbidades também se associou ao desfecho (OR = 1,86; IC95%: 1,12–3,10; p = 0,015). A anorexia, apesar de significativa na análise bivariada, apresentou associação inversa na regressão, reduzindo a chance de COVID-19 (OR = 0,32; IC95%: 0,21–0,49; p < 0,001) (Tabela 4).

**Tabela 4:** Regressão logística das variáveis demográficas, clínicas, sinais e sintomas associados à ocorrência da COVID-19 na população pediátrica hospitalizada (n = 486) – Londrina, PR, Brasil, 2023.

Variáveis	OR ajustado	IC95%	Valor de p^‡^
**Local de internação**			
Pronto-socorro	2,78	1,33–5,84	0,007
Unidade de Terapia Intensiva	2,52	1,54–4,16	0,000
Internação pediátrica	1,00		
**Uso de corticoide**			
Sim	3,37	2,05–5,53	<0,001
Não	1,00		
**Anorexia**			
Sim	0,32	0,21–0,49	<0,001
Não			
**Coriza**			
Sim	2,25	1,43–3,54	<0,001
Não			
**Comorbidades**			
Sim	1,86	1,12–3,10	0,015
Não			

Legenda: OR – Odds Ratio; IC95% – Intervalo de Confiança de 95%

^‡^teste qui-quadrado de Pearson.

## DISCUSSÃO

Este estudo forneceu evidências sobre os fatores predisponentes e associações com a ocorrência da COVID-19 na população pediátrica hospitalizada. Entre os indicadores utilizados, na análise multivariada, o local de internação (pronto-socorro e UTI), uso de corticoide, ter coriza e comorbidades foram os mais fortemente associados à chance de as crianças terem COVID-19.

A análise dos resultados identificou que, independentemente do grupo, houve um maior acometimento da COVID-19 em crianças do sexo masculino. Pesquisas precursoras em Wuhan, na China, no início da pandemia, validam este dado, e evidências apontam que os pacientes do sexo masculino tendem a ter uma maior incidência da doença e, quando comparados aos pacientes do sexo feminino, há uma maior probabilidade de morte ou de desenvolver formas graves da doença^(6,13)^.

Entre as crianças diagnosticadas com COVID-19, observou-se que 74,5% tinham menos de 10 anos de idade. Tais dados corroboram o estudo que apresenta uma análise retrospectiva de infecções por SARS-CoV-2 com 2.135 pacientes, prevalecendo a idade média de 7 anos, sendo essa população susceptível a ser contaminada pelo vírus^(14)^.

O local de internação se destacou como fator relevante, pois crianças internadas no pronto-socorro apresentaram 2,78 vezes mais chance de diagnóstico de COVID-19, enquanto aquelas internadas na UTI tiveram chance 2,52 vezes maior, em comparação à internação em enfermaria pediátrica. Esses achados podem estar relacionados ao perfil clínico inicial dos pacientes, à maior rotatividade e aglomeração de leitos nessas unidades, e à maior exposição a pacientes sintomáticos, especialmente no início da pandemia, quando havia escassez de equipamentos de proteção individual e protocolos ainda estavam em construção^(15)^.

A mediana encontrada referente ao tempo de internação foi de dois dias, resultado próximo ao encontrado na literatura^(16)^, sendo que, quanto maior o tempo de internação, maiores os riscos para os pacientes, devido à exposição a agentes infecciosos durante a hospitalização. Como a população pediátrica tem direito a acompanhantes, sua família também fica exposta a contrair doenças e, do mesmo modo, propagá-las^(17)^. O perfil do paciente pediátrico é diverso e inerente às etapas de desenvolvimento, e existem algumas características comuns em relação às demandas de cuidados hospitalares a serem atribuídas. Este dado pode ser de grande relevância para auxiliar nas rotinas hospitalares e reorganização de serviços pediátricos, quando necessário, juntamente com as informações sobre o aspecto clínico dos pacientes^(16)^.

Importante ressaltar que 64,2% das crianças hospitalizadas não fizeram uso de suporte ventilatório. Estudos enfatizam a discussão sobre utilização de suporte ventilatório com intenção de evitar danos futuros e aumento de internação hospitalar^(18)^. Sabe-se que crianças são susceptíveis a desenvolver infecções nas vias respiratórias em decorrência das suas qualidades anatômicas, fisiológicas e imunológicas, e quando necessário a utilização de suporte ventilatório, o processo de desmame ventilatório compreende em 40% o tempo de internação^(19)^.

O uso de antimicrobiano e corticoide foi amplamente empregado nas crianças investigadas. Nesta amostra, a análise indicou que crianças que utilizaram antimicrobianos apresentaram 58% menos chance de serem casos de COVID-19. Esse uso pode ser justificado pela tentativa de barrar infecção no trato respiratório, uma vez que, no princípio pandêmico, o conhecimento sobre o vírus era limitado, e se tratando de infecções respiratórias, há possibilidade de haver infecção mista ou infecção bacteriana secundária^(20)^. Outra explicação é a cobertura empírica para possível superinfecção no trato respiratório e que seja adquirida em hospital^(21)^.

O uso de corticoide também se associou ao maior risco de COVID-19, com aumento de 3,37 vezes na chance de diagnóstico, quando comparado ao controle. Contudo, o uso de corticoide muitas vezes é empregado em infecções de vias aéreas, devido ao seu efeito anti-inflamatório, à redução de replicação viral e à regulação da expressão de genes da enzima conversora de angiotensina 2^(22)^.

Nesse sentido, avaliando a descrição dos sinais e sintomas da população pediátrica observados no estudo, pôde-se verificar que a presença de febre esteve associada a um aumento de 90% na chance de diagnóstico de COVID-19. Febre esteve presente na maior parte das crianças do grupo caso, sendo essa uma das queixas mais comuns em clínicas e hospitais infantis^(23)^, além de um sinal referido isoladamente em cerca de 20 a 30% das consultas pediátricas. Além disso, a tosse foi verificada em ambos os grupos, o que vem de encontro com o estudo que destacou também a tosse como o principal sintoma identificado em 83% das crianças^(24)^.

Os sintomas de tosse, coriza, padrão respiratório não eupneico e saturação de oxigênio de 90 a 95% apresentaram associação inversa com o diagnóstico de COVID-19, estando associados a uma menor chance de ocorrência desse desfecho na população estudada. No geral, em grande parte dos sinais e sintomas de casos de síndrome gripal, a tosse e a coriza foram apontadas como sintomatologia inicial, considerados sintomas clássicos das infecções aéreas, podendo ser seguidas de outros sintomas. Desse modo, fármacos são amplamente utilizados, devido ao incômodo gerado e à alteração da qualidade de vida a curto prazo^(25)^.

Outro fator importante identificado foi a presença de comorbidades, que aumentou em 1,86 vezes a chance de COVID-19. Crianças com condições clínicas preexistentes, como doenças pulmonares crônicas ou antecedentes de prematuridade, podem apresentar maior susceptibilidade à infecção e evolução clínica menos favorável. De acordo com o *The International Study of Asthma and Allergies in Childhood*, um protocolo internacional validado e padronizado que favorece os estudos sobre asma e doenças alérgicas, apontou-se que o Brasil é um país com alta prevalência de doenças pulmonares, em especial asma e rinite alérgica^(26)^.

A prematuridade ainda causa impactos na morbimortalidade dos recém-nascidos e sequelas permanentes no desenvolvimento das crianças. No Brasil, nos anos de 2011 a 2021, foram notificados cerca de 31.625.722 nascidos-vivos; desses, 3.503.085 eram prematuros, ou seja, com uma prevalência de 11%^(27)^. Tais sequelas se refletem no futuro das crianças, sendo um fator de risco para crianças quando em contato com agressores virais respiratórios. Entretanto, nossos achados revelam que 76,3% das crianças não tinham outras doenças relacionadas. No entanto, a presença de comorbidades esteve associada a um aumento de 86% na chance de COVID-19. Possível explicação é que possuir comorbidade aumenta a chance de evoluir com prognóstico desfavorável^(15)^.

Entre as crianças diagnosticadas com COVID-19, observou-se maior frequência de indivíduos que se autodeclararam pardos, pretos, amarelos ou indígenas, em comparação às crianças brancas, e a análise revelou que essas crianças apresentaram 55% mais chance de compor o grupo de casos, indicando uma associação estatisticamente significativa entre raça/cor e ocorrência de COVID-19 na população estudada. Estudo de caso-controle e revisão sistemática com metánalise informam que grupos raciais e étnicos (negros, latinos e hispânicos) são afetados de uma forma desproporcional, podendo ser por questões econômicas, desigualdades e condições de saúde análogas^(14)^.

Observou-se que o uso de máscara de oxigênio e cateter nasal esteve mais frequentemente associado ao grupo controle. Esses achados sugerem que o uso desses suportes ventilatórios pode estar relacionado a quadros respiratórios de outras etiologias. O cateter nasal é um dos métodos de terapia não invasiva mais eficazes, devido à capacidade de evitar aerossolização e contaminação cruzada, além de ser confortável^(28)^, e quando fornecido o suporte ventilatório, há redução de esforço do organismo, auxiliando na melhora do paciente e restabelecendo os níveis normais de oxigênio necessários para a saúde^(29)^.

Em face dos resultados, observou-se que o local de internação (pronto-socorro e UTI), a presença de comorbidades, o uso de corticoide e o sintoma de coriza permaneceram significativamente associados ao diagnóstico de COVID-19. Crianças hospitalizadas no pronto-socorro apresentaram 2,78 vezes mais chance de diagnóstico, aquelas internadas em UTI, 2,52 vezes, e as que utilizaram corticoide, 3,37 vezes. Além disso, a presença de coriza esteve associada a um aumento de 2,25 vezes na chance de compor o grupo de casos. Esses achados evidenciam a complexidade da assistência à saúde de crianças acometidas pela COVID-19, e reforçam a necessidade de ampliação das estratégias de prevenção, diagnóstico precoce e cuidados terapêuticos específicos para esse grupo populacional.

As limitações deste estudo incluem o caráter retrospectivo, que impossibilitou a observação direta da criança, e o fato de ter sido conduzido em um único hospital de ensino, o que pode limitar a generalização dos resultados. Outra limitação está relacionada ao não cegamento dos pacientes pertencentes ao grupo controle pelo pesquisador principal e a informações incompletas em banco de dados, acarretando inconsistência e, consequentemente, exclusão dos dados.

## CONCLUSÃO

Entre os fatores associados à ocorrência da COVID-19 em menores de 14 anos hospitalizados, o local de internação (pronto-socorro e UTI), uso de corticoide, ter coriza e comorbidades foram os mais fortemente associados ao diagnóstico da doença. Os resultados deste estudo avançam no conhecimento ao fornecer informações importantes sobre sinais e sintomas apresentados pelas crianças durante a hospitalização pela COVID-19, bem como os fatores associados a essas ocorrências.

## DISPONIBILIDADE DE DADOS

Todo o conjunto de dados que dá suporte aos resultados deste estudo foi publicado no próprio artigo.

# Translation: Factores asociados a la aparición de COVID-19 en la población pediátrica en el ámbito hospitalario: un estudio de casos y controles

**DOI:** 10.1590/1980-220X-REEUSP-2025-0211es

**Authors:** Denise Desconsi, Juliane Pagliari Araujo, Marcela Demitto Furtado, Rosângela Aparecida Pimenta, Adriana Valongo Zani

**Affiliations:** 1 Universidade Estadual de Londrina, Londrina, PR, Brasil.; 2 Universidade Estadual de Maringá, Maringá, PR, Brasil.

**Keywords:** Niño, Hospitalización, COVID-19, Pandemias, Estudios de Casos y Controles

## Abstract

**Objetivo:**

Analizar los factores asociados a la incidencia de COVID-19 en la población pediátrica en un entorno hospitalario.

**Método:**

Estudio de casos y controles pareados, realizado con historias clínicas de niños menores de 14 años. Se consideró como población caso a la población pediátrica con resultado positivo para COVID-19, y como población control a la población pediátrica con resultado negativo para COVID-19. Por cada caso, se utilizó un control, totalizando 486 historias clínicas. Se realizaron análisis descriptivo, análisis bivariado y regresión logística.

**Resultados:**

Las variables asociadas con la ocurrencia de COVID-19 fueron: niños de raza mixta, negros, asiáticos e indígenas, ingreso a urgencias y unidad de cuidados intensivos, uso de mascarilla y catéter de oxígeno, antimicrobianos, y corticosteroides. Fiebre, anorexia, patrón respiratorio no eupneico con saturación entre 90% y 95%, tos, rinorrea y comorbilidad se asociaron con el desenlace.

**Conclusión:**

Este estudio avanza al proporcionar información sobre los factores asociados con COVID-19 en la población hospitalizada menor de 14 años, incluyendo el lugar de hospitalización, anorexia, rinorrea, comorbilidad y uso de corticosteroides.

## INTRODUCCIÓN

El COVID-19 se descubrió en el año 2019, y su agente etiológico es el SARS-CoV-2, presentando una alta transmisibilidad con propagación a escala mundial, lo que llevó a la Organización Mundial de la Salud a declarar la pandemia de COVID-19 en marzo de 2020^(1)^.

La transmisión de la COVID-19 se produce rápidamente de persona a persona, ya sea por contacto directo o por exposición a pequeñas gotas de secreciones infectadas en superficies^(2)^. En el contexto pediátrico, los niños de todas las edades pueden contraer la enfermedad, como se demostró en una cohorte multicéntrica que evaluó a 582 niños con COVID-19, en la que se verificó que el grupo de edad más afectado es el de 10 a 18 años^(3)^.

Las características clínicas y epidemiológicas cambian constantemente, y la presentación de los síntomas de la COVID-19 en los niños puede ser amplia y variada, pudiendo ser asintomática o sintomática, además de presentar casos graves como dificultad respiratoria aguda grave^(4)^.

En una revisión sistemática de la literatura, se observa que de los 342 niños positivos para COVID-19, 51 eran asintomáticos^(5)^. Los datos obtenidos en Wuhan, China, al inicio de la pandemia, indican que de los 171 niños hospitalizados, con una edad media de entre 6 y 7 años y con prueba positiva para COVID-19, 27 eran asintomáticos^(6)^. Sin embargo, se puede observar que las manifestaciones de la enfermedad actúan de manera diferente en adultos y niños, y a menudo son similares a las de otros virus respiratorios, presentando un cuadro clínico común, con fiebre, tos y cefalea^(7)^. Además, en una revisión de alcance, se identificó la presencia de síntomas gastrointestinales presentados por la población pediátrica con COVID-19^(8)^.

La escasez de datos sobre las afecciones relacionadas, los síntomas presentados y la COVID-19 en niños menores de 14 años indica que hay lagunas que deben llenarse^(9)^. Este estudio debe ayudar a comprender la variabilidad de la enfermedad y contribuir al desempeño de la enfermería, que desempeña un papel destacado en la prevención y el cuidado de la enfermedad.

Por lo tanto, es necesaria la identificación temprana de los síntomas en los niños, ya que hay muchos factores implicados que pueden favorecer el contagio y el agravamiento de la enfermedad, además de disminuir la transmisión del virus^(8)^. Para ello, se plantea la siguiente pregunta: ¿qué factores están asociados a la aparición de la COVID-19 en la población pediátrica en el contexto hospitalario?

El objetivo de este estudio es analizar los factores asociados a la aparición de la COVID-19 en la población pediátrica en el contexto hospitalario.

## MÉTODO

### Tipo de Estudio

Se trata de un estudio de casos y controles pareados, descrito de acuerdo con la herramienta*STrengthening the Reporting of OBservational studies in Epidemiology*.

### Lugar del Estudio

El estudio se llevó a cabo en los sectores de urgencias pediátricas, Unidad de Cuidados Intensivos (UCI) y hospitalización pediátrica de un hospital universitario (HU) público situado en la región norte del estado de Paraná, Brasil. La institución es un referente en el tratamiento de la COVID-19. Este estudio consideró como lugares de hospitalización la sala de urgencias, la enfermería y la UTI pediátrica, dada la necesidad de mantener a los niños hospitalizados en estos lugares debido a la demanda. La confirmación de la COVID-19 se realizó en el lugar de hospitalización del niño.

### Población

La población del estudio incluyó (n = 486) historias clínicas de pacientes pediátricos menores de 14 años atendidos en el HU y que ingresaron en el hospital entre junio de 2020 y diciembre de 2022. Este periodo se consideró el pico pandémico de COVID-19 en Brasil y, para las hospitalizaciones pediátricas, el hospital del estudio considera la población neonatal de 0 a 28 días y la infantil, de 29 días a 14 años.

### Criterios de Selección y Definición de la Muestra

Se consideró caso a la población pediátrica con COVID-19 positivo. Para el grupo control, se consideró la población pediátrica con síntomas, pero que dio negativo para COVID-19, realizándose, para cada caso, un control^(10)^, obteniéndose así 243 casos y 243 controles, lo que suma un total de 486 expedientes/pacientes. Por lo tanto, se utilizaron todos los casos de pacientes pediátricos positivos para COVID-19 en el período investigado, sin necesidad de realizar un cálculo muestral. Para realizar el pareamiento de las muestras entre el caso y el control, se optó por la afinidad de los datos entre individuos, teniendo en cuenta las características, pero no aquellas que estaban siendo investigadas. Así, para definir el grupo control en referencia al grupo de casos, se observaron las variables de sexo y edad, buscando similitudes entre las poblaciones.

Se excluyó a los niños que no tenían exámenes de laboratorio confirmatorios para COVID-19. Para identificar la muestra, se utilizaron dos hojas de cálculo proporcionadas por el núcleo de vigilancia de salud de la institución hospitalaria: la primera es una hoja de cálculo de pacientes con prueba de COVID-19 positiva; y la segunda es una hoja de cálculo de pacientes con prueba de COVID-19 negativa. La Figura 1 presenta el diagrama de flujo de selección de los expedientes médicos/pacientes y muestra los grupos de casos y controles, teniendo en cuenta los criterios de inclusión y exclusión.

**Figura 1:** Selección de las historias clínicas/pacientes para el estudio. Londrina, Paraná, Brasil, 2023.

### Variables del Estudio

La variable de resultado fue la COVID-19. La positividad se confirmó mediante pruebas de transcripción inversa, seguidas de reacción en cadena de la polimerasa, antígeno, serología, inmunoglobulina G y M rápida registradas en el historial clínico de los pacientes y en la hoja de cálculo COVID-19.

Las variables de exposición fueron sexo, edad, raza/color/etnia, ciudad de origen, ingreso hospitalario, lugar de hospitalización, tiempo de hospitalización, soporte ventilatorio, uso de antimicrobianos y uso de corticoides. Las variables relacionadas con los signos y síntomas fueron la presencia de fiebre, anorexia, reflujo, náuseas, vómitos, diarrea, sangrado intestinal, dolor abdominal, patrón respiratorio, saturación de oxígeno, tos, coriza y comorbilidades. Las variables analizadas en este estudio se basaron en estudios realizados anteriormente^(5,11,12)^.

### Recopilación de Datos

La información demográfica, clínica y relacionada con los signos y síntomas de los pacientes se extrajo de los expedientes médicos y se introdujo en dos hojas de cálculo electrónicas por separado. La recopilación de datos se llevó a cabo entre el 10 de abril de 2023 y el 24 de mayo de 2023, a través de*Medview* ^®^ *,*el sistema informático de expedientes médicos electrónicos del hospital.

### Análisis de Datos

Los datos se analizaron con el paquete estadístico*Statistical Package for the Social Sciences*, versión 26.0. El análisis descriptivo incluyó la descripción de la muestra estudiada, con énfasis en las características demográficas y clínicas. Se midieron las frecuencias absolutas y relativas de las variables de interés. Para medir la asociación entre el resultado y las variables de exposición, se utilizaron análisis bivariados y multivariados.

En el análisis bivariado, se aplicó la prueba de chi-cuadrado de Pearson, seleccionándose para la siguiente etapa las variables que presentaron alfa < 0,20, punto de corte recomendado para análisis exploratorios de factores asociados. Se calcularon las razones de probabilidad (o*odds ratio*[OR]) brutas y sus intervalos de confianza del 95% (IC del 95%).

Los modelos múltiples se obtuvieron mediante regresión logística binaria, por el método*backward*, es decir, el orden de retirada de las variables de exposición en el modelo viene determinado por el mayor valor de significación. Las variables con alfa < 0,05 permanecieron en el modelo. Se calcularon las OR ajustadas y sus IC95%. La calidad del ajuste se midió mediante la prueba de Hosmer-Lesmeshow, en la que cuanto mayor es el valor de p, mejor es el ajuste. Se consideró alfa < 0,05 como estadísticamente significativo.

### Aspectos Éticos

El estudio fue aprobado por el Comité de Ética en Investigación con Seres Humanos de una institución pública de enseñanza, bajo el Certificado de Presentación para Apreciación Ética n.º 31528920.9.0000.5231 y el Dictamen n.º 4.415.191. No hubo contacto directo con los pacientes, por lo que se dispensó el uso del Término de Consentimiento Libre e Informado.

## RESULTADOS

La muestra estuvo compuesta por 486 expedientes/pacientes, siendo 243 casos confirmados de COVID-19 y 243 controles. Se observó que el 61,3% de los niños, independientemente del grupo, eran varones, menores de 10 años (74,5% de los casos y 77% de los controles) y de raza blanca (60,9% de los casos y 70,9% de los controles), y fueron derivados desde otros servicios hospitalarios (85,6% de los casos y 86,4% de los controles). En cuanto al lugar de hospitalización, los niños con COVID-19 fueron hospitalizados principalmente en urgencias (47.7%), mientras que los del grupo control se encontraban en su mayoría en la sala de pediatría (66.3%). La mediana del tiempo de hospitalización fue de tres días para los casos y de un día para los controles.

En cuanto al soporte ventilatorio, se observó que el 74.5% de los niños con COVID-19 no necesitaron ningún dispositivo, mientras que en el grupo control este porcentaje fue del 53.9%. En cuanto al uso de antimicrobianos y corticoides, ambos fueron más frecuentes en el grupo control, administrándose al 70.4% y al 82.7% de los niños, respectivamente. Entre los casos de COVID-19, el uso de estos medicamentos fue menor, ya que el 49.8% de los niños recibieron antimicrobianos y el 48.1%, corticoides.

Los síntomas más prevalentes fueron fiebre (60.1% en los casos y 65.8% en el control), tos (73.3% en los casos y 55.1% en el control) y coriza (56.4% en los casos y 74.5% en el control). La anorexia fue más frecuente en el grupo de casos (56.4%) que en el de control (36.6%). Las enfermedades pulmonares crónicas y la prematuridad fueron las comorbilidades más comunes (Tabla 1).

**Tabla 1:** Características demográficas y clínicas de la población pediátrica hospitalizada (n = 486) – Londrina, PR, Brasil, 2023.

Variables	Caso n (%)	Control n (%)
**Sexo**		
Mujer	94 (38,7)	94 (38,7)
Hombres	149 (61,3)	149 (61,3)
**Edad**		
< 10 años	181 (74,5)	187 (77,0)
≥ 10 años	62 (25,5)	56 (23,0)
Mediana (IIC)	3,00 (9,00)	4,00 (8,00)
Mínimo-Máximo	0–14	0–14
**Raza/color**		
Blanco	148 (60,9)	172 (70,9)
Marrón, negro, amarillo, indígena	95 (39,1)	71 (29,1)
**Ingreso hospitalario**		
Derivados	208 (85,6)	210 (86,4)
Búsqueda espontánea	35 (14,4)	33 (13,6)
**Lugar de hospitalización**		
Servicio de urgencias	116 (47,7)	46 (18,9)
Unidad de cuidados intensivos	21 (8,7)	36 (18,8)
Ingreso pediátrico	106 (43,6)	161 (66,3)
**Tiempo de hospitalización (días)**		
≥ 7 días	29 (11,9)	30 (12,3)
< 7 días	214 (88,1)	213 (87,7)
Mediana (IIC)	3,00 (3,00)	1,00 (3,00)
Mínimo-Máximo	1–35	1–30
**Soporte ventilatorio**		
Mascarilla	8 (3,3)	25 (10,3)
Catéter	35 (14,4)	65 (26,7)
Intubación orotraqueal	19 (7,8)	22 (9,1)
Ninguna	181 (74,5)	131 (53,9)
**Uso de antimicrobianos**		
Sí	121 (49,8)	171 (70,4)
No	122 (50,2)	72 (29,6)
**Uso de corticosteroides**		
Sí	117 (48,1)	201 (82,7)
No	126 (51,9)	42 (17,3)
**Presencia de fiebre**		
Sí	146 (60,1)	160 (65,8)
No	97 (39,9)	83 (34,2)
**Anorexia**		
Sí	137 (56,4)	89 (36,6)
No	106 (43,6)	154 (63,4)
**Reflujo gástrico**		
Sí	4 (1,6)	6 (2,5)
No	239 (98,4)	237 (97,5)
**Náuseas**		
Sí	25 (10,3)	21 (8,6)
No	218 (89,7)	222 (91,4)
**Vómitos**		
Sí	58 (23,9)	46 (18,9)
No	185 (76,1)	197 (81,1)
**Diarrea**		
Sí	35 (14,4)	38 (15,6)
No	208 (85,6)	205 (84,4)
**Hemorragia intestinal**		
Sí	8 (3,3)	8 (3,3)
No	235 (96,7)	235 (96,7)
**Dolor abdominal**		
Sí	44 (18,1)	28 (11,5)
No	199 (81,9)	215 (85,5)
**Patrón respiratorio**		
Disneico	110 (45,3)	67 (27,6)
Taquipnéico	9 (3,7)	17 (7,0)
Eupneicas	124 (51,0)	159 (65,4)
**Saturación de oxígeno**		
< 90%	20 (8,2)	22 (9,1)
90 a 95%	97 (39,9)	59 (24,3)
> 95%	126 (51,9)	162 (66,6)
**Tos**		
Sí	178 (73,3)	134 (55,1)
No	65 (26,7)	109 (44,9)
**Goteo nasal**		
Sí	137 (56,4)	181 (74,5)
No	106 (43,6)	62 (25,5)
**Comorbilidades**		
Prematuridad	14 (5,8)	20 (8,2)
Enfermedad pulmonar crónica	35 (14,4)	14 (5,8)
Encefalopatía hipóxico-isquémica	10 (4,1)	2 (0,8)
Trastornos endocrinos	13 (5,3)	2 (0,8)
Obesidad	2 (0,8)	3 (1,2)
Sin comorbilidades	169 (69,6)	202 (83,2)

Leyenda: IIC – intervalo intercuartílico.

En el análisis bivariante, algunas variables mostraron una asociación estadísticamente significativa con la aparición de COVID-19. Los niños clasificados como pardos, negros, amarillos o indígenas tenían 1,55 veces más probabilidades de ser diagnosticados de COVID-19 en que los niños blancos (OR = 1,55; IC 95%: 1,1–2,27; p = 0,022). El ingreso en urgencias aumentó casi cuatro veces la probabilidad de COVID-19, en comparación con el ingreso pediátrico (OR = 3,83; IC 95%: 2,52–5,83; p < 0,001). El uso de máscara de oxígeno (OR = 0,23), catéter (OR = 0,38), antimicrobianos (OR = 0,42) y corticosteroides (OR = 0,19) se asoció con una menor probabilidad de diagnóstico de COVID-19, todos con p < 0,001 (Tabla 2).

**Tabla 2:** Asociaciones entre factores demográficos y clínicos y la ocurrencia de COVID-19 en la población pediátrica hospitalizada (n = 486) – Londrina, PR, Brasil, 2023.

Variables	COVID-19		Valor p*	OR bruta (IC 95%)
	Caso n (%)	Control n (%)		
**Edad**				
< 10 años	181 (74,5)	187 (77,0)	0,526	0,87 (0,57–1,32)
≥ 10 años	62 (25,5)	56 (23,0)		
Mediana (IIC)	3 (8,00)			
Mínimo-máximo	0–14			
**Sexo**				
Mujer	94 (38,7)	94 (38,7)	1,00	1,00 (0,69–1,44)
Varón	149 (61,3)	149 (61,3)		
**Raza/color**				
Blanco	148 (60,9)	172 (70,8)		
Marrón, negro, amarillo, indígena	95 (39,1)	71 (29,2)	0,022	1,55 (1,1–2,27)
**Ingreso hospitalario**				
Derivados	209 (86,0)	209 (86,0)	1,00	1,00 (0,77–1,29)
Búsqueda espontánea	34 (14,0)	34 (14,0)		
**Lugar de hospitalización**				
Servicio de urgencias	116 (47,7)	46 (18,9)	<0,001	3,83 (2,52–5,83)
Unidad de cuidados intensivos	21 (8,6)	36 (14,8)	0,688	0,89 (0,49–1,60)
Ingreso pediátrico	106 (43,7)	161 (66,3)		
**Tiempo de hospitalización (días)**				
≥ 7 días	29 (11,9)	30 (12,3)	0,890	1,04 (0,60–1,79)
< 7 días	214 (88,1)	213 (87,7)		
Mediana (IIC)	2 (3,00)			
Mínimo-Máximo	1–35			
**Soporte ventilatorio**				
Mascarilla	8 (3,3)	25 (10,3)	<0,001	0,23 (0,10–0,53)
Catéter	35 (14,4)	65 (26,7)	<0,001	0,38 (0,24–0,62)
Tubo	19 (7,8)	22 (9,1)	0,078	0,62 (0,32–1,20)
Aire ambiente	181 (74,5)	131 (53,9)		
**Uso de antimicrobianos**				
Sí	121 (49,8)	171 (70,4)	<0,001	0,42 (0,28–0,61)
No	122 (50,2)	72 (29,6)		
**Uso de corticosteroides**				
Sí	117 (48,1)	201 (82,7)	<0,001	0,19 (0,13–0,24)
No	126 (51,9)	42 (17,3)		

Leyenda: *Prueba chi-cuadrado de Pearson; OR: *Odds Ratio*; IC95%: intervalo de confianza del 95%; IIC: intervalo intercuartílico.

Con respecto a los signos y síntomas, los niños con COVID-19 tenían más probabilidades de presentar fiebre (OR = 1,90; IC 95%: 1,31–2,76; p = 0,001) y anorexia (OR = 2,24; IC 95%: 1,55–3,22; p < 0,001) que el grupo de control. Por otro lado, síntomas como la rinorrea (OR = 0,44), la tos (OR = 0,52) y el patrón respiratorio no eupneico (OR = 0,45) se asociaron más con el grupo control. La presencia de comorbilidades también fue más común entre el grupo control (32,9% caso y 14,4% control; OR = 0,34; p < 0,001), sugiriendo una menor asociación con COVID-19 (Tabla 3).

**Tabla 3:** Asociaciones entre signos y síntomas asociados a la ocurrencia de COVID-19 en la población pediátrica hospitalizada (n = 486) – Londrina, PR, Brasil, 2023.

Variables	COVID-19		Valor p*	OR bruta (IC 95%)
	Caso n (%)	Control n (%)		
**Presencia de fiebre**				
Sí	171 (70,4)	135 (55,6)	0,001	1,90 (1,31–2,76)
No	72 (29,6)	108 (44,4)		
**Anorexia**				
Sí	137 (56,4)	89 (36,6)	<0,001	2,24 (1,55–3,22)
No	106 (43,6)	154 (63,4)		
**Reflujo gástrico**				
Sí	5 (2,1)	5 (2,1)	1,00	1,00 (0,29–3,50)
No	238 (97,9)	238 (97,9)		
**Náuseas**				
Sí	25 (10,3)	21 (8,6)	0,535	1,21 (0,66–2,23)
No	218 (89,7)	222 (91,4)		
**Vómito**				
Sí	48 (19,8)	56 (23,0)	0,376	0,82 (0,53–1,27)
No	195 (80,2)	187 (77,0)		
**Diarrea**				
Sí	41 (16,9)	32 (13,2)	0,253	1,34 (0,81–2,21)
No	202 (83,1)	211 (86,8)		
**Hemorragia intestinal**				
Sí	7 (2,9)	9 (3,7)	0,611	0,77 (0,28–2,10)
No	236 (97,1)	234 (96,3)		
**Dolor abdominal**				
Sí	42 (17,3)	30 (12,3)	0,125	1,48 (0,89–2,46)
No	201 (82,7)	213 (87,7)		
**Patrón respiratorio**				
No eupneico	78 (32,1)	125 (51,4)	<0,001	0,45 (0,31–0,64)
Eupneico	165 (67,9)	118 (48,6)		
**Saturación de oxígeno**				
< 90%	21 (8,6)	21 (8,6)	0,309	0,71 (0,37–1,37)
90 a 95%	54 (22,2)	102 (42,0)	<0,001	0,38 (0,25–0,57)
> 95%	168 (69,1)	120 (49,4)		
**Tos**				
Sí	138 (56,8)	174 (71,6)	0,001	0,52 (0,36–0,76)
No	105 (43,2)	69 (28,4)		
**Goteo nasal**				
Sí	137 (56,4)	181 (74,5)	<0,001	0,44 (0,30–0,65)
No	106 (43,6)	62 (25,5)		
**Comorbilidades**				
Sí	35 (14,4)	80 (32,9)	<0,001	0,34 (0,22–0,52)
No	208 (85,6)	163 (67,1)		

Leyenda: *Prueba chi-cuadrado de Pearson; OR: *Odds Ratio*; IC95%: Intervalo de Confianza del 95%; IIC: IIC intercuartílico.

En el análisis multivariante, algunas variables permanecieron asociadas al diagnóstico de COVID-19 en la población pediátrica. Los niños hospitalizados en urgencias tenían 2,78 veces más probabilidades de padecer COVID-19 (IC 95%: 1,33–5,84; p = 0,007), y los hospitalizados en la UCI tenían 2,52 veces más probabilidades (IC 95%: 1,54–4,16; p < 0,001), en comparación con la hospitalización pediátrica.

El uso de corticosteroides aumentó la probabilidad de COVID-19 en 3,37 veces (IC 95%: 2,05-5,53; p < 0,001), y la presencia de coriza en 2,25 veces (IC 95%: 1,43–3,54; p < 0,001). Tener comorbilidades también se asoció con el resultado (OR = 1,86; IC 95%: 1,12–3,10; p = 0,015). La anorexia, aunque significativa en el análisis bivariante, mostró una asociación inversa en la regresión, reduciendo la probabilidad de COVID-19 (OR = 0,32; IC 95%: 0,21–0,49; p < 0,001) (Tabla 4).

**Tabla 4:** Regresión logística de variables demográficas y clínicas, signos y síntomas asociados a la ocurrencia de COVID-19 en la población pediátrica hospitalizada (n = 486) – Londrina, PR, Brasil, 2023.

Variables	OR ajustado	IC 95%	Valor p^‡^
**Lugar de hospitalización**			
Servicio de urgencias	2,78	1,33–5,84	0,007
Unidad de cuidados intensivos	2,52	1,54–4,16	0,000
Ingreso pediátrico	1,00		
**Uso de corticosteroides**			
Sí	3,37	2,05–5,53	<0,001
No	1,00		
**Anorexia**			
Sí	0,32	0,21–0,49	<0,001
No			
**Goteo nasal**			
Sí	2,25	1,43–3,54	<0,001
No			
**Comorbilidades**			
Sí	1,86	1,12–3,10	0,015
No			

Leyenda: OR – Odds Ratio; IC 95% – Intervalo de Confianza del 95%

^‡^Prueba chi-cuadrado de Pearson.

## DISCUSIÓN

Este estudio proporcionó evidencia sobre los factores predisponentes y las asociaciones con la aparición de COVID-19 en la población pediátrica hospitalizada.

Entre los indicadores utilizados en el análisis multivariante, el lugar de hospitalización (urgencias y UCI), el uso de corticoides, la rinitis y las comorbilidades fueron los más fuertemente asociados con la probabilidad de que los niños tuvieran COVID-19.

El análisis de los resultados identificó que, independientemente del grupo, hubo una mayor incidencia de COVID-19 en los niños varones. Las investigaciones precursoras realizadas en Wuhan, China, al inicio de la pandemia, validan este dato, y las evidencias apuntan a que los pacientes varones tienden a tener una mayor incidencia de la enfermedad y, en comparación con las pacientes mujeres, hay una mayor probabilidad de muerte o de desarrollar formas graves de la enfermedad^(6,13)^.

Entre los niños diagnosticados con COVID-19, se observó que el 74,5% tenía menos de 10 años. Estos datos corroboran el estudio que presenta un análisis retrospectivo de las infecciones por SARS-CoV-2 con 2135 pacientes, con una edad media de 7 años, siendo esta población susceptible de ser contagiada por el virus^(14)^.

El lugar de hospitalización se destacó como un factor relevante, ya que los niños hospitalizados en urgencias tenían 2,78 veces más probabilidades de ser diagnosticados con COVID-19, mientras que los hospitalizados en la UCI tenían 2,52 veces más probabilidades, en comparación con los hospitalizados en la sala pediátrica. Estos hallazgos pueden estar relacionados con el perfil clínico inicial de los pacientes, la mayor rotación y aglomeración de camas en estas unidades y la mayor exposición a pacientes sintomáticos, especialmente al inicio de la pandemia, cuando había escasez de equipo de protección individual y los protocolos aún se estaban elaborando^(15)^.

La mediana encontrada en relación con el tiempo de hospitalización fue de dos días, un resultado similar al encontrado en la literatura^(16)^, y cuanto mayor es el tiempo de hospitalización, mayores son los riesgos para los pacientes, debido a la exposición a agentes infecciosos durante la hospitalización. Dado que la población pediátrica tiene derecho a acompañantes, sus familiares también están expuestos a contraer enfermedades y, del mismo modo, a propagarlas^(17)^. El perfil del paciente pediátrico es diverso e inherente a las etapas de desarrollo, y existen algunas características comunes en relación con las demandas de cuidados hospitalarios que se deben asignar. Este dato puede ser de gran relevancia para ayudar en las rutinas hospitalarias y la reorganización de los servicios pediátricos, cuando sea necesario, junto con la información sobre el aspecto clínico de los pacientes^(16)^.

Es importante destacar que el 64,2% de los niños hospitalizados no utilizaron asistencia respiratoria. Los estudios enfatizan el debate sobre el uso de la asistencia respiratoria con la intención de evitar daños futuros y el aumento de las hospitalizaciones^(18)^. Se sabe que los niños son susceptibles de desarrollar infecciones de las vías respiratorias debido a sus características anatómicas, fisiológicas e inmunológicas, y cuando es necesario el uso de asistencia respiratoria, el proceso de destete respiratorio comprende el 40% del tiempo de hospitalización^(19)^.

El uso de antimicrobianos y corticoides fue ampliamente empleado en los niños investigados. En esta muestra, el análisis indicó que los niños que utilizaron antimicrobianos tuvieron un 58% menos de probabilidades de ser casos de COVID-19. Este uso puede justificarse por el intento de detener la infección en el tracto respiratorio, ya que, al inicio de la pandemia, el conocimiento sobre el virus era limitado y, en el caso de las infecciones respiratorias, existe la posibilidad de que haya una infección mixta o una infección bacteriana secundaria^(20)^. Otra explicación es la cobertura empírica para una posible sobreinfección en el tracto respiratorio y que se adquiera en el hospital^(21)^.

El uso de corticoides también se asoció con un mayor riesgo de COVID-19, con un aumento de 3,37 veces en la probabilidad de diagnóstico, en comparación con el control. Sin embargo, el uso de corticoides se emplea a menudo en infecciones de las vías respiratorias, debido a su efecto antiinflamatorio, a la reducción de la replicación viral y a la regulación de la expresión de los genes de la enzima convertidora de angiotensina 2^(22)^.

En este sentido, al evaluar la descripción de los signos y síntomas de la población pediátrica observados en el estudio, se pudo verificar que la presencia de fiebre se asoció con un aumento del 90% en la probabilidad de diagnóstico de COVID-19. La fiebre estuvo presente en la mayoría de los niños del grupo de casos, siendo esta una de las quejas más comunes en clínicas y hospitales infantiles^(23)^, además de un signo referido de forma aislada en alrededor del 20 al 30% de las consultas pediátricas. Además, se observó tos en ambos grupos, lo que concuerda con el estudio que también destacó la tos como el síntoma principal identificado en el 83% de los niños^(24)^.

Los síntomas de tos, coriza, patrón respiratorio no eupneico y saturación de oxígeno del 90 al 95% presentaron una asociación inversa con el diagnóstico de COVID-19, estando asociados a una menor probabilidad de que se produjera este desenlace en la población estudiada. En general, en la mayoría de los signos y síntomas de los casos de síndrome gripal, la tos y la rinitis se señalaron como sintomatología inicial, considerados síntomas clásicos de las infecciones aéreas, que pueden ir seguidos de otros síntomas. Por lo tanto, los medicamentos se utilizan ampliamente, debido a las molestias que generan y a la alteración de la calidad de vida a corto plazo^(25)^.

Otro factor importante identificado fue la presencia de comorbilidades, que aumentó en 1,86 veces la probabilidad de COVID-19. Los niños con afecciones clínicas preexistentes, como enfermedades pulmonares crónicas o antecedentes de prematuridad, pueden presentar una mayor susceptibilidad a la infección y una evolución clínica menos favorable. Según el Estudio Internacional sobre Asma y Alergias en la Infancia, un protocolo internacional validado y estandarizado que favorece los estudios sobre el asma y las enfermedades alérgicas, se señaló que Brasil es un país con una alta prevalencia de enfermedades pulmonares, en especial asma y rinitis alérgica^(26)^.

La prematuridad sigue teniendo repercusiones en la morbilidad y mortalidad de los recién nacidos y secuelas permanentes en el desarrollo de los niños. En Brasil, entre los años 2011 y 2021, se notificaron alrededor de 31 625 722 nacidos vivos; de ellos, 3 503 085 eran prematuros, es decir, con una prevalencia del 11%^(27)^. Estas secuelas se reflejan en el futuro de los niños, siendo un factor de riesgo para ellos cuando entran en contacto con agresores virales respiratorios. Sin embargo, nuestros hallazgos revelan que el 76.3% de los niños no tenían otras enfermedades relacionadas. No obstante, la presencia de comorbilidades se asoció con un aumento del 86% en la probabilidad de contraer COVID-19. Una posible explicación es que tener comorbilidades aumenta la probabilidad de evolucionar con un pronóstico desfavorable^(15)^.

Entre los niños diagnosticados con COVID-19, se observó una mayor frecuencia de individuos que se autodeclararon morenos, negros, amarillos o indígenas, en comparación con los niños blancos, y el análisis reveló que estos niños tenían un 55% más de probabilidades de formar parte del grupo de casos, lo que indica una asociación estadísticamente significativa entre la raza/color y la aparición de COVID-19 en la población estudiada. Un estudio de casos y controles y una revisión sistemática con metaanálisis informan que los grupos raciales y étnicos (negros, latinos e hispanos) se ven afectados de manera desproporcionada, lo que puede deberse a cuestiones económicas, desigualdades y condiciones de salud análogas^(14)^.

Se observó que el uso de mascarillas de oxígeno y catéteres nasales se asoció con mayor frecuencia al grupo control. Estos hallazgos sugieren que el uso de estos soportes ventilatorios puede estar relacionado con cuadros respiratorios de otras etiologías. El catéter nasal es uno de los métodos de terapia no invasiva más eficaces, debido a su capacidad para evitar la aerosolización y la contaminación cruzada, además de ser cómodo^(28)^, y cuando se proporciona el soporte ventilatorio, se reduce el esfuerzo del organismo, lo que ayuda a la mejoría del paciente y restablece los niveles normales de oxígeno necesarios para la salud^(29)^.

A la luz de los resultados, se observó que el lugar de hospitalización (urgencias y UCI), la presencia de comorbilidades, el uso de corticoides y el síntoma de coriza seguían estando significativamente asociados al diagnóstico de COVID-19. Los niños hospitalizados en urgencias tenían 2,78 veces más probabilidades de ser diagnosticados, los ingresados en la UCI, 2,52 veces, y los que utilizaron corticoides, 3,37 veces. Además, la presencia de coriza se asoció con un aumento de 2,25 veces en la probabilidad de formar parte del grupo de casos. Estos hallazgos evidencian la complejidad de la atención médica de los niños afectados por COVID-19 y refuerzan la necesidad de ampliar las estrategias de prevención, diagnóstico precoz y cuidados terapéuticos específicos para este grupo poblacional.

Las limitaciones de este estudio incluyen su carácter retrospectivo, que imposibilitó la observación directa del niño, y el hecho de que se llevó a cabo en un solo hospital universitario, lo que puede limitar la generalización de los resultados. Otra limitación está relacionada con la falta de cegamiento de los pacientes pertenecientes al grupo control por parte del investigador principal y con la información incompleta en la base de datos, lo que provocó inconsistencias y, en consecuencia, la exclusión de datos.

## CONCLUSIÓN

Entre los factores asociados a la aparición de la COVID-19 en menores de 14 años hospitalizados, el lugar de ingreso (urgencias y UCI), el uso de corticoides, la rinitis y las comorbilidades fueron los más fuertemente asociados al diagnóstico de la enfermedad. Los resultados de este estudio amplían el conocimiento al proporcionar información importante sobre los signos y síntomas que presentan los niños durante la hospitalización por COVID-19, así como los factores asociados a estos casos.

## DISPONIBILIDAD DE DATOS

El conjunto completo de datos que respalda los hallazgos de este estudio está disponible en el mismo artículo.
